# Endobronchial Intratumoral Immuno- and Gene Therapies in Lung Cancer: Mechanisms of Local Delivery, Systemic Immune Effects, and Global Feasibility

**DOI:** 10.3390/ijms27156988

**Published:** 2026-08-04

**Authors:** Mihai Olteanu, Gabriela Marina Andrei, Ramona Cioboată, Virginia Maria Rădulescu

**Affiliations:** 1 Department of Internal Medicine-Pneumology, Faculty of Medicine, University of Medicine and Pharmacy from Craiova, 200349 Craiova, Romania; mihai.olteanu@umfcv.ro; 2 Department of Medical Informatics and Biostatistics, Faculty of Medicine, University of Medicine and Pharmacy of Craiova, 200349 Craiova, Romania; virginia.radulescu@umfcv.ro

**Keywords:** lung cancer, intratumoral therapy, endobronchial ultrasound, oncolytic virus, gene therapy, tumour microenvironment, abscopal effect, bronchoscopic delivery, immunotherapy, NSCLC

## Abstract

Lung cancer remains the leading cause of cancer-related mortality worldwide, and durable benefit from immune checkpoint inhibitors is limited by immune exclusion, microenvironmental immunosuppression, and toxicity. This narrative translational review evaluates whether endobronchial intratumoral delivery of immuno- and gene-based therapies can transform bronchoscopic access into a therapeutic platform linking local tumour intervention with systemic antitumour immunity. We synthesise evidence on endobronchial ultrasound, electromagnetic navigation bronchoscopy, and robotic-assisted bronchoscopy, together with local checkpoint blockade, cytokine and mRNA-lipid nanoparticle constructs, oncolytic virotherapy, dendritic-cell approaches, viral and non-viral gene transfer, and enzyme- or metabolite-based strategies. Current clinical evidence remains preliminary, consisting mainly of Phase I trials, small prospective cohorts, and case-based signals, with feasibility and safety observations but no completed randomised trial demonstrating efficacy against standard-of-care systemic therapy. The most plausible candidates are patients with advanced or recurrent NSCLC, bronchoscopically accessible lesions, inadequate response to systemic immunotherapy, and immune-excluded or locally immunosuppressed tumours. Biomarkers such as PD-L1, tumour mutational burden, CD8+ infiltration, tertiary lymphoid structures, radiomics, and circulating tumour DNA require validation. This review integrates delivery technology, immune mechanisms, statistical evidence appraisal, biomarker limitations, AI-guided planning, and global feasibility.

## 1. Introduction

Lung cancer is both the most lethal malignancy on Earth and, in the past decade, one of the fields in which therapeutic innovation has been most rapid—a paradox that reflects not inadequate scientific ambition but the profound biological complexity of a disease that has proved, time and again, more resourceful than the treatments designed to contain it. Lung cancer accounts for approximately 1.8 million deaths annually worldwide, more than colorectal, breast, and prostate cancer combined, and remains the leading cause of cancer-related mortality across both sexes in the majority of countries where data are reliably collected [1]. Projections to 2050 suggest continued increases in absolute incidence driven by ageing populations, persistent tobacco exposure in low- and middle-income settings, and rising rates of never-smoker adenocarcinoma [2,3]. Approximately 85% of all lung cancers are non-small cell in histology, with the majority diagnosed at advanced, unresectable stages [1].

The development of immune checkpoint inhibitors (ICIs) targeting the PD-1/PD-L1 axis has unquestionably altered the natural history of advanced NSCLC for a subset of patients, converting a disease with a median overall survival measured in months into one in which durable, years-long remissions are achievable [4,5]. Yet the therapeutic gains have been unevenly distributed. Across unselected NSCLC populations, approximately 60–70% of patients fail to achieve a durable response to checkpoint blockade, whether due to primary refractoriness at treatment initiation or to acquired resistance after an initial response [6,7].

Three biological mechanisms account for most of this failure. First, systemic checkpoint inhibitors can amplify existing anti-tumour T-cell responses but cannot generate them: a tumour that has excluded T cells entirely presents the immunological equivalent of a locked room—and no key the ICI possesses will open it [8,9]. Second, the immunosuppressive tumour microenvironment (TME)—maintained by myeloid-derived suppressor cells, regulatory T cells, TGF-β, IL-10, and adenosine pathways—actively extinguishes nascent immune responses before they reach therapeutic scale [4,5]. Third, dose-limiting immune-related adverse events (irAEs)—colitis, pneumonitis, and endocrinopathy—restrict dose escalation and combination strategies in a clinically significant proportion of patients [6]. These three barriers define the pharmacological boundaries of systemic checkpoint blockade and motivate a qualitatively different therapeutic strategy.

Endobronchial intratumoral delivery of immuno- and gene therapeutic agents represents precisely such a strategy. By depositing therapeutic agents directly within the TME, intratumoral delivery achieves drug concentrations orders of magnitude higher than those accessible by intravenous administration—concentrations sufficient to trigger immunogenic cell death (ICD), activate the cGAS–STING innate immune pathway, prime polyclonal tumour-antigen-specific T-cell responses, and—under the right biological conditions—generate systemic anti-tumour immunity against distant, non-injected lesions through the abscopal mechanism [10,11,12]. The endobronchial route provides anatomical access to this target through an established clinical platform: bronchoscopy and endobronchial ultrasound (EBUS) are routinely performed across tertiary and many secondary oncology centres without percutaneous intervention, ionising radiation guidance, or traversal of critical intrathoracic structures [13,14]. Advances in electromagnetic navigation bronchoscopy (ENB) and robotic-assisted platforms have further extended this reach to peripheral lesions previously inaccessible by this route [10,11].

In this review, we examine the scientific and clinical landscape of endobronchial intratumoral immuno- and gene therapy in lung cancer. We describe the bronchoscopic delivery platforms and their pharmacokinetic characteristics; systematically review local immunotherapy modalities (checkpoint inhibitor injection, cytokine-based and mRNA-LNP approaches, oncolytic virotherapy, and dendritic cell vaccines) and gene therapy strategies (viral vectors, nanoparticle systems, KRAS/EGFR-targeting constructs); analyse the molecular mechanisms linking locoregional intervention to systemic immunity—including ICD, the cGAS–STING cascade, the abscopal effect, and tertiary lymphoid structure induction; synthesise available clinical trial data with explicit attention to statistical evidence quality; and address the critical question of global feasibility across healthcare systems of varying resource levels. Statistical considerations are integrated throughout rather than relegated to separate subsections, consistent with the view that appraisal of evidence quality is inseparable from the evidence itself.

## 2. Methods of Literature Selection

This review represents a narrative translational synthesis of the available literature rather than a formal systematic review or meta-analysis. The objective was to integrate mechanistic, translational, and clinical evidence related to endobronchial intratumoral immuno- and gene therapies in lung cancer, with particular emphasis on bronchoscopic delivery platforms, local immune activation, systemic antitumour effects, and feasibility of clinical implementation.

To avoid an overly diffuse synthesis, the review was deliberately organised around four linked translational questions: which bronchoscopic platforms can provide reproducible intratumoral access; which therapeutic classes have sufficient mechanistic or early clinical rationale for local delivery; which biological mechanisms plausibly connect local intervention to systemic antitumour immunity; and what methodological, biomarker, regulatory, and feasibility barriers must be addressed before clinical implementation. Topics outside this framework, including systemic immunotherapy not directly related to intratumoral delivery, general lung cancer management, and non-bronchoscopic local therapies without relevance to endobronchial access, were not treated as independent areas of review.

Literature searches were performed using PubMed/MEDLINE, Scopus, Web of Science, and ClinicalTrials.gov databases. Search terms included combinations of: “lung cancer”, “NSCLC”, “intratumoral therapy”, “endobronchial”, “bronchoscopy”, “EBUS”, “electromagnetic navigation bronchoscopy”, “robotic bronchoscopy”, “gene therapy”, “oncolytic virus”, “mRNA”, “STING”, “abscopal effect”, “tumour microenvironment”, and “intratumoral immunotherapy”. Priority was given to publications from 2015 to 2026, although seminal earlier studies were included where historically or mechanistically relevant.

Eligible sources included early-phase clinical trials, prospective cohort studies, translational investigations, mechanistic experimental studies, consensus guidelines, and high-impact reviews relevant to the biological and clinical rationale of intratumoral therapy in lung cancer. Ongoing clinical trials were identified through ClinicalTrials.gov and incorporated when directly relevant to currently developing therapeutic platforms.

Given the heterogeneity of study designs, endpoints, and therapeutic platforms, no formal quantitative meta-analysis was performed. Instead, evidence was synthesised narratively, with explicit attention to methodological limitations, statistical power, confidence intervals, biomarker validation, and translational applicability.

## 3. Bronchoscopic Delivery Systems for Intratumoral Access

### 3.1. Anatomical Rationale and Platform Overview

The endobronchial route provides a uniquely privileged anatomical corridor for intratumoral therapy. The tracheobronchial tree extends in intimate proximity to a substantial proportion of primary pulmonary malignancies, hilar lymph nodes, and mediastinal recurrences—many of which are inaccessible to percutaneous approaches without traversing critical structures such as major vessels, the pleura, or the pericardium [10,11]. Unlike transthoracic injection, bronchoscopic delivery eliminates pneumothorax risk, requires no ionising radiation guidance, and permits repeated treatment under conscious sedation. The ability to combine diagnostic sampling, molecular profiling, and therapeutic delivery in a single bronchoscopic event is the most operationally distinctive advantage of this route [13].

**Figure 1:**
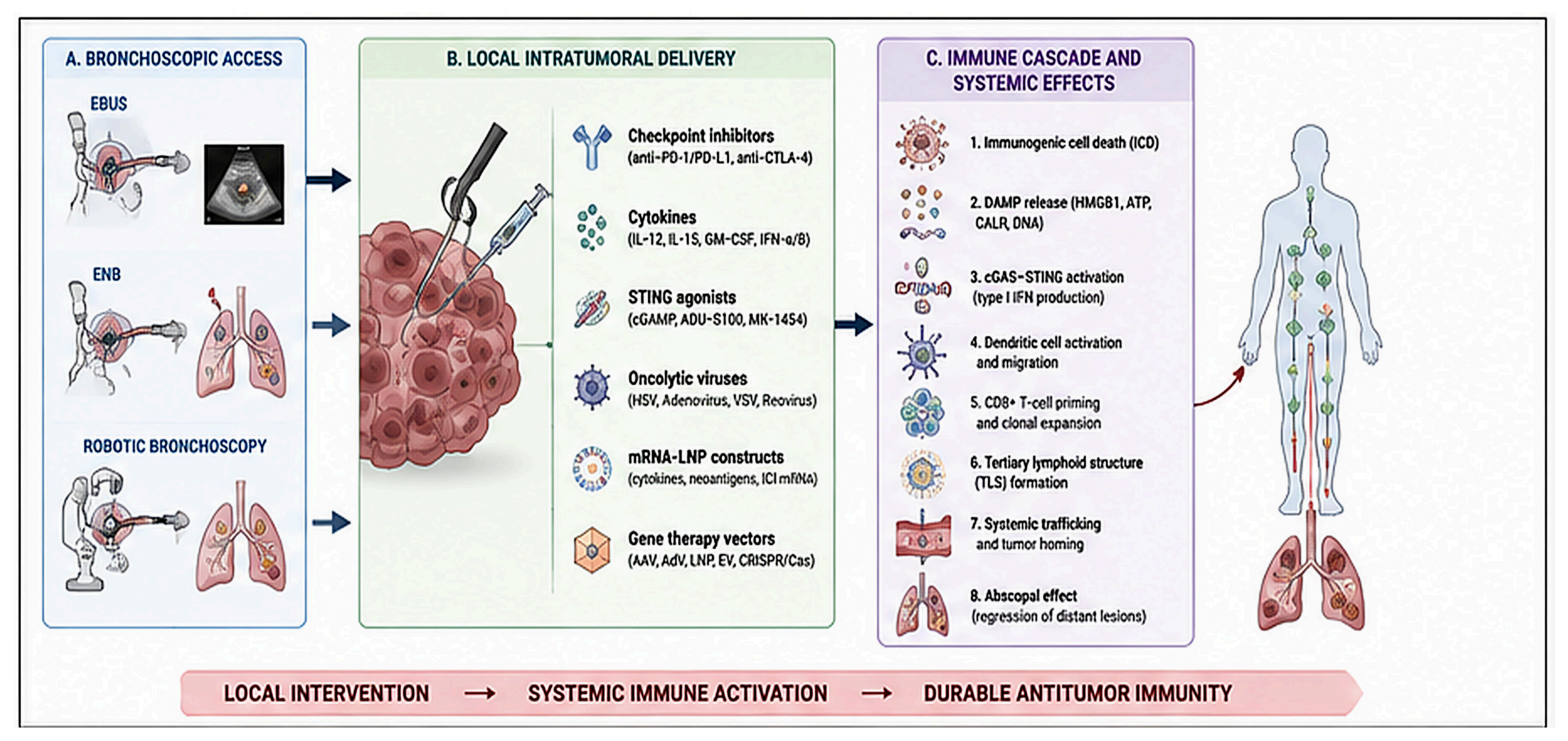
Conceptual transition from bronchoscopic intratumoral delivery to systemic antitumour immune activation.

Three platforms currently provide bronchoscopic access for intratumoral delivery. The conceptual transition from bronchoscopic local delivery to systemic antitumour immune activation is summarised in Figure 1. Endobronchial ultrasound (EBUS), integrating a 5–12 MHz curvilinear transducer, enables real-time transbronchial needle placement with continuous imaging guidance and achieves staging sensitivity of 88–93% for mediastinal lymph nodes [10,11,14]. Electromagnetic navigation bronchoscopy (ENB) extends reach to peripheral lesions using CT-derived virtual roadmaps, though diagnostic yields of 59–80% reflect dependence on lesion size, CT bronchus sign, and registration accuracy between preprocedural imaging and intraprocedural anatomy [11]. Robotic-assisted bronchoscopy (RAB)—principally the Monarch, Ion, and Archimedes platforms—combines shape-sensing navigation with computer-controlled catheter drive and intraoperative cone-beam CT (CBCT) fluoroscopy for tool-in-lesion confirmation [15,16,17].

Multisite real-world data from more than 2000 RAB procedures report a diagnostic sensitivity of approximately 85% for peripheral lesions [18]; confidence intervals for this estimate were not uniformly reported across contributing sites, and a component of learning-curve improvement is embedded in the aggregate figure. Multi-focal lesions across different lobes may be accessed within a single anaesthetic event—a practical advantage that reduces cumulative procedural risk and positions RAB as the platform of choice for patients with synchronous or multi-focal pulmonary disease [15]. The comparative procedural and translational characteristics of the principal bronchoscopic delivery platforms are summarised in Table 1.

### 3.2. Delivery Devices and Drug Distribution

The physical characteristics of intratumoral drug distribution are substantially shaped by the instrumentation used. Conventional EBUS needles produce a single depot injection that distributes along the path of least tissue resistance—typically through fracture planes rather than uniformly through interstitial spaces—resulting in heterogeneous spatial coverage [13]. The Blowfish microinjector catheter (Galvanise Therapeutics), designed specifically for endobronchial use, administers the drug via a series of discrete injections in a near-circumferential spatial distribution, maximising target coverage [19,20].

In the only multicentre prospective safety study of endobronchial intratumoral chemotherapy, Yarmus et al. [19] enrolled 23 patients with NSCLC and malignant central airway obstruction, of whom 19 received Blowfish-delivered nanoparticulate paclitaxel at a mean of 3.4 injections per session. No device-related serious adverse events were recorded. This encouraging safety signal must be contextualised statistically: with n = 19 and zero observed events, the exact binomial upper 95% confidence limit for the true adverse event rate (Clopper-Pearson) is 17.6%—meaning an adverse event rate of up to 1 in 6 patients cannot be excluded from these data alone [19]. Larger, prospectively registered safety cohorts are a methodological priority before device-specific adverse event rates can be characterised with regulatory precision. Device-specific performance characterisation is increasingly recognised as a prerequisite for the rational design of intratumoral therapy trials [21].

A further safety issue that requires explicit consideration is the theoretical risk that intratumoral manipulation may facilitate tumour dissemination. Although endobronchial delivery avoids pleural traversal and may therefore reduce some risks associated with transthoracic approaches, the act of needle insertion and intratumoral pressure injection can still mechanically disrupt tumour architecture, increase interstitial pressure, displace viable tumour cells along tissue planes, or promote leakage into adjacent vascular or lymphatic channels. In addition, the local inflammatory and stromal remodelling induced by some intratumoral agents may, under insufficiently controlled biological conditions, create a microenvironment that is not exclusively antitumoural. Current endobronchial intratumoral studies are too small and too heterogeneous to quantify this risk reliably, and the absence of reported procedure-related dissemination should not be interpreted as definitive evidence of absence. Future trials should therefore prospectively monitor potential dissemination-related endpoints, including new lesions along the bronchoscopic access route, rapid emergence of regional nodal or distant metastases after injection, serial circulating tumour DNA dynamics, and imaging-defined patterns of progression in injected versus non-injected lesions. This issue is particularly relevant when repeated injections, high-volume delivery, highly vascular tumours, or necrotic and friable lesions are considered.

### 3.3. Computational Modelling of Intratumoral Pharmacokinetics

A three-dimensional pharmacodynamic model of EBUS-guided intratumoral cisplatin delivery, calibrated against serum concentration data from 32 NSCLC patients, correctly predicted radiographic clinical response in 72% of cases—with superior accuracy for adenocarcinomas (80%) over squamous cell carcinomas (62.5%) [22]. From a statistical standpoint, two limitations temper these results. First, with n = 32, the exact 95% CI for 72% accuracy spans approximately 53–87% (Clopper-Pearson), meaning the true model performance could be substantially lower than the point estimate. Second, the model was calibrated and evaluated on the same patient cohort without independent external validation—a design that systematically inflates apparent predictive performance through overfitting [22].

Despite these constraints, the model established a conceptually important finding: apportioning the total drug dose across multiple spatially distributed injection sites consistently increases the volume of tumoricidal drug exposure, providing a mechanistic basis for multi-site injection strategies. It further identified a power-law relationship between tumour volume and the minimum effective drug dose, supporting dose individualisation based on pre-procedural CT metrics—a framework increasingly amenable to integration with AI-assisted intraprocedural navigation [23].

## 4. Endobronchial Intratumoral Immunotherapy

**Figure 2:**
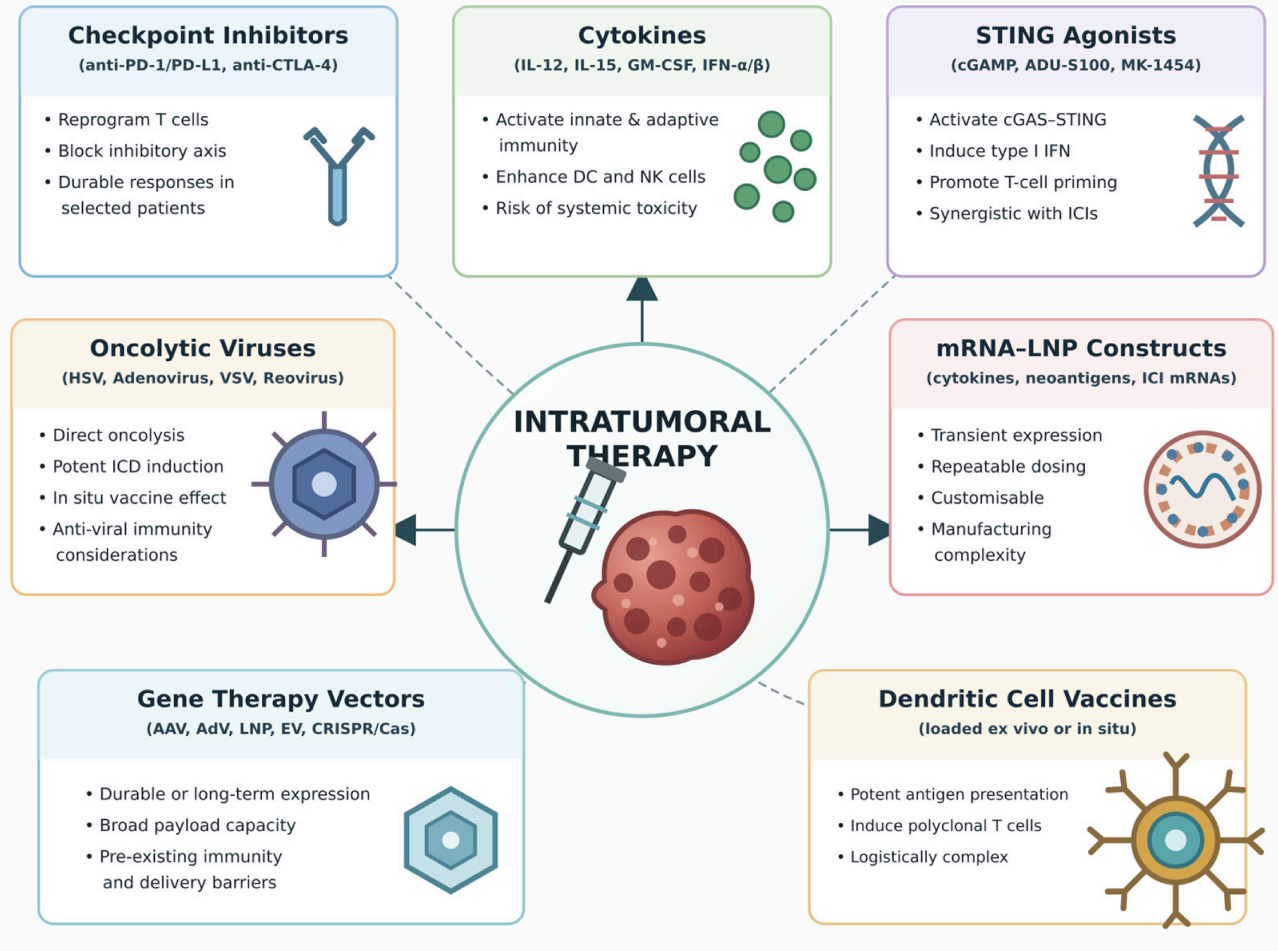
Therapeutic ecosystem of bronchoscopic intratumoral immunotherapy in lung cancer.

The principal therapeutic classes currently explored for bronchoscopic intratumoral delivery are conceptually summarised in Figure 2.

### 4.1. Local Checkpoint Inhibitor Injection

The rationale for intratumoral rather than intravenous administration of checkpoint inhibitors rests on a pharmacokinetic calculation of benefit and risk. Local injection generates drug concentrations within the TME that are orders of magnitude higher than those achievable by systemic dosing, while—in principle—limiting the systemic drug exposure that drives immune-related adverse events such as colitis, pneumonitis, and endocrinopathy [24,25]. Whether local administration truly confines immunological effects locally, or whether the downstream systemic immune activation that constitutes therapeutic success also restores systemic irAE risk, is a question clinical trial data have not yet definitively resolved [25].

At the molecular engineering level, nanoparticulated anti-PD-1 antibody formulations have been specifically designed to optimise local retention after intratumoral injection. Mujahid et al. [26] demonstrated that PEG-coated anti-PD-1 nanoparticles exhibited significantly prolonged intratumoural residence compared to free antibody—reducing systemic leakage, extending local target engagement, and achieving superior tumour growth inhibition in preclinical models at a fraction of the systemic dose required for equivalent effect. A clinical illustration of the potential of local checkpoint delivery was reported by Liu et al. [27], who described a patient with advanced lung adenocarcinoma refractory to systemic therapy who achieved a sustained complete response following repeated intratumoral injections of tirelizumab via tracheoscopy. While a single case report carries no statistical weight by definition, it functions as a proof-of-concept signal—mechanistically consistent with the conversion of an immunologically excluded tumour to an immune-responsive state—that warrants prospective investigation in a powered cohort.

### 4.2. Cytokine-Based Approaches and mRNA-LNP Platforms

The history of systemic cytokine therapy in oncology is, in large part, a history of ambition constrained by toxicity. High-dose interleukin-2 (IL-2) achieved durable complete responses in a minority of patients with metastatic melanoma and renal cell carcinoma, but at the cost of capillary leak syndrome requiring ICU-level monitoring, with treatment-related mortality of approximately 1–4% in early series [28]. Intratumoral administration rewrites this equation: cytokines delivered locally can achieve immunostimulatory concentrations within the TME without the systemic drug exposure that drives endothelial toxicity.

IL-12 has emerged as the cytokine of greatest current interest for intratumoral delivery. It drives IFN-γ production by T cells and NK cells, potentiates cytotoxic lymphocyte differentiation, promotes dendritic cell maturation, and counteracts the immunosuppressive TGF-β and IL-10 signalling that dominates the NSCLC microenvironment [28,29]. Di Trani et al. [29] demonstrated that intratumoral injection of IL-12-encoding mRNA specifically targeted to tumour-infiltrating CSF1R+ myeloid cells and PD-L1+ cells achieved potent anti-tumour effects in preclinical models without measurable systemic IL-12 elevation—a pharmacological separation between local efficacy and systemic toxicity that systemic IL-12 administration has consistently failed to achieve.

Horton et al. [28] engineered non-toxic IL-12 and IL-2 variant proteins with attenuated receptor binding kinetics that reduce systemic cytokine syndrome while preserving local immunostimulatory activity, demonstrating in orthotopic lung cancer models that their combination overcame ICI resistance and generated CD8+ T-cell-dependent tumour regression at non-injected sites. Li et al. [30] extended this combinatorial principle further, showing that intratumoral LNP-encoded IL-12/IL-7/IFN-α cocktail mRNA generated sustained cytokine expression for 48–72 h post-injection with tumour regression in 70% of treated animals—underscoring the value of multi-cytokine strategies over single-agent approaches.

The most clinically advanced mRNA-LNP construct for intratumoral use is mRNA-2752, which simultaneously encodes OX40 ligand, IL-23, and IL-36γ [31,32,33]. In the Phase I dose-escalation trial (NCT03739931), mRNA-2752 alone and in combination with durvalumab demonstrated pharmacodynamic induction of an immunostimulatory gene expression signature in paired tumour biopsies—confirming target engagement at the tumour site [32,33]. A mechanistically distinct approach using inhalable extracellular vesicle-delivered IL-12 mRNA achieved intratumoural cytokine expression with systemic immune activation in orthotopic murine NSCLC models, median survival extended 2.8-fold, and 40% complete tumour clearance without bronchoscopic access—though its applicability to central or mediastinal disease remains to be established [34].

### 4.3. Enzyme- and Metabolite-Based Intratumoral Strategies

Beyond checkpoint inhibitors, cytokines, mRNA constructs, and viral platforms, enzyme- and metabolite-based strategies represent an emerging class of intratumoral interventions that target the metabolic architecture of the tumour microenvironment rather than a single immune checkpoint or antigenic pathway. Their rationale is particularly relevant to solid tumours in which high glycolytic flux, lactate accumulation, extracellular acidosis, hypoxia, and nutrient competition contribute to T-cell dysfunction and resistance to immune checkpoint blockade. In this context, metabolite-directed intratumoral therapy aims either to deplete immunosuppressive metabolites, such as lactate, or to generate cytotoxic and immunostimulatory products directly within the tumour bed.

Lactate oxidase-based therapy illustrates this principle. Cao et al. developed lactate oxidase nanocapsules designed to reduce intratumoral lactate concentrations while generating immunostimulatory hydrogen peroxide, thereby enhancing T-cell-mediated antitumour immunity and improving the efficacy of cancer immunotherapy in preclinical models [35]. This approach is conceptually attractive for lung cancer because metabolically active, hypoxic NSCLC lesions frequently combine lactate-driven immune suppression with poor effector T-cell penetration. However, its application to endobronchial intratumoral delivery remains translational rather than clinically established. Key unresolved issues include local control of hydrogen peroxide generation, heterogeneity of lactate availability across tumour regions, the possibility of off-target oxidative injury to adjacent bronchial or vascular structures, and the need for pharmacodynamic biomarkers confirming metabolic reprogramming at the injected site.

Glucose oxidase (GOx)-based systems operate through a complementary metabolic mechanism. GOx catalyses the oxidation of β-D-glucose into gluconic acid and hydrogen peroxide, thereby combining glucose depletion, oxidative stress generation, oxygen consumption, and local acidification within the tumour microenvironment [36]. These features have been exploited in multimodal nanomedicine platforms that combine GOx with chemodynamic, photothermal, starvation-like, or immune-activating strategies. For bronchoscopic intratumoral therapy, GOx-based constructs could theoretically be formulated as locally retained nanoreactors or injectable enzyme carriers, allowing high substrate turnover within the tumour while minimising systemic enzymatic exposure. Nevertheless, this therapeutic class is not yet supported by clinical endobronchial lung cancer data, and its future evaluation should include not only conventional response endpoints but also intratumoral glucose and lactate dynamics, oxidative stress markers, immune-cell infiltration, circulating tumour DNA kinetics, and comparative response patterns between injected and non-injected lesions. Enzyme- and metabolite-based intratumoral therapies should therefore be viewed as candidate modulators of the metabolic–immune interface, with potential complementarity to checkpoint blockade, cytokine delivery, and gene-based platforms, but with substantial safety and delivery questions that remain to be addressed.

### 4.4. Oncolytic Virotherapy

Oncolytic viruses occupy a unique therapeutic niche: they kill tumour cells through viral replication and lysis and simultaneously trigger ICD, releasing tumour antigens, DAMPs, and pathogen-associated molecular patterns into a locally inflamed microenvironment [37,38]. The MEM-288 platform (Memgen, NCT05076760) employs a replication-selective adenoviral vector encoding interferon-β (IFN-β) and CD40 ligand—a dual design in which IFN-β drives innate immune activation and MHC class I upregulation while CD40L engagement of dendritic cells promotes cross-presentation and T-cell priming [39]. The Phase I trial includes an NSCLC expansion cohort; no dose-limiting toxicities were reported at the first two dose levels.

A parallel programme evaluating oncolytic virus RT-01 in extensive-stage SCLC (NCT05205421) [40] targets a histology characterised by very high mutational burden and initial ICI responsiveness—creating conditions in which ICD-driven immune activation could theoretically outpace the immune evasion mechanisms that characterise SCLC progression. The bronchoscopic route confers a specific biological advantage for adenovirus-based platforms: respiratory epithelial cells and airway-adjacent tumour cells express the coxsackievirus-adenovirus receptor at substantially higher levels than percutaneously accessible peripheral sites, potentially enhancing viral transduction efficiency and making endobronchial delivery the preferred route for this class of agents [37].

### 4.5. Dendritic Cell Vaccines and CCL21-Mediated Intratumoral Priming

Dendritic cell-based immunotherapy has been historically limited by the failure of systemically administered DCs to traffic to and persist within the TME in sufficient numbers to sustain T-cell priming [41,42]. Intratumoral delivery eliminates this trafficking requirement: DCs are deposited within the immunological context where their activity is needed, exposed to the full antigenic complexity of the tumour in situ. Ingels et al. [43] demonstrated that neoantigen-targeted DC vaccination—loading DCs with patient-specific tumour neoantigens identified by whole-exome sequencing—generated long-lived antigen-specific T-cell responses in advanced NSCLC patients, with vaccine-induced clonotypes persisting in peripheral blood for up to 12 months and detectable in post-treatment tumour biopsies. Median progression-free survival was 8.6 months in this single-arm cohort. The absence of a concurrent control arm—an inherent limitation of this study design—means that this figure cannot be directly compared to alternative therapies without introducing selection bias.

The CCL21-modified DC approach developed by Lee et al. [44] takes a mechanistically distinct direction: rather than loading DCs with predefined antigens, CCL21-gene-modified DCs are injected directly into the tumour to establish a chemokine gradient that recruits naïve T cells, plasmacytoid DCs, and additional antigen-presenting cells to the injection site—transforming the tumour into a tertiary lymphoid priming hub in situ. Post-treatment biopsies demonstrated a median 3.2-fold increase in CD8+ T-cell density (*p* = 0.012, Wilcoxon), dose-dependent upregulation of PD-L1 (an adaptive immune resistance signal indicating active immune engagement rather than constitutive immune evasion), and elevated intratumoural IFN-γ without systemic cytokine release. This PD-L1 upregulation provided the immunological rationale for the subsequent combination trial of intratumoral CCL21-DC plus systemic pembrolizumab (NCT03546361) [45]—predicated on the hypothesis that CCL21-DC converts a PD-L1-negative, ICI-resistant tumour into a PD-L1-positive, ICI-sensitive state.

### 4.6. Statistical Appraisal of the Intratumoral Immunotherapy Evidence Base

The evidence base for endobronchial intratumoral immunotherapy is, without exception, composed of Phase I dose-escalation studies, small cohort case series, and Phase I/II expansion cohorts—study designs powered to identify dose-limiting toxicities, not to establish efficacy. Phase I 3+3 designs, with cohort sizes of 3–6 patients per dose level and total enrolments of 15–40 patients, have essentially no statistical power to detect differences in objective response rates, progression-free survival, or overall survival [11,24]. An observed response in a Phase I trial is a signal, not evidence—it warrants further investigation in an appropriately powered study but cannot, by itself, establish population-level efficacy.

Response assessment in intratumoral immunotherapy trials carries an additional complexity absent from conventional cytotoxic therapy: pseudoprogression. The initial influx of immune cells into the tumour can transiently increase lesion size on imaging, producing apparent progression by RECIST 1.1 criteria that represents an evolving therapeutic response. The frequency of true pseudoprogression in systemic ICI recipients is approximately 4–10% across tumour types; for intratumoral delivery—where local immune activation is particularly rapid and concentrated—this frequency may be higher. The iRECIST criteria, requiring confirmation of apparent progression at a second imaging timepoint no less than four weeks later, provide a more appropriate assessment framework for intratumoral immunotherapy trials and should be adopted as the standard in future study protocols [24,25].

Single-arm study designs—which dominate this landscape—introduce susceptibility to selection bias, regression-to-the-mean in small convenience samples, and incomparability with historical controls, which is exacerbated by secular trends in supportive care. These limitations do not invalidate the mechanistic insights generated by existing studies, but they define the evidential ceiling below which adoption in clinical practice is not warranted and above which a randomised, adequately powered Phase II trial is required.

## 5. Gene Therapy Strategies for Intratumoral Delivery in Lung Cancer

### 5.1. From Tumour Suppressor Restoration to Immunostimulatory Gene Delivery

Gene therapy, in its original conception, was a discipline of correction—the ambition to repair or replace defective genes in order to restore normal cellular function. Applied to cancer, this corrective logic found its earliest clinical expression in the restoration of tumour suppressor genes silenced by somatic mutation. Three decades of clinical experience have refined that original vision: tumour biology is rarely correctable by a single genetic intervention, and what has emerged from that evolution is a more sophisticated therapeutic logic that uses gene delivery not merely to correct but to instruct—delivering immunostimulatory transgenes, oncogene-silencing constructs, and molecular switches that reprogram the TME rather than simply repairing individual cells [46,47,48,49]. For lung cancer specifically, the bronchoscopic intratumoral route maximises local transduction efficiency, limits vector biodistribution to non-target tissues and reduces the neutralising antibody responses that systemic administration provokes.

The landscape of viral and non-viral delivery systems currently in clinical or preclinical development for intratumoral bronchoscopic delivery is summarised in Table 2. Properties including cargo capacity, immunogenicity, repeat-dosing tolerance, and clinical development stage are compared across adenoviral vectors, AAV, oncolytic viruses, lipid nanoparticle-mRNA (LNP-mRNA), polymeric nanoparticles, PEGylated siRNA peptides, extracellular vesicles, and CRISPR-Cas9 platforms [50,51,52,53]. Rather than describing each system in parallel prose—which would largely duplicate the table—the following subsections focus on the clinical evidence and mechanistic insights that the tabular format cannot efficiently capture.

**Figure 3:**
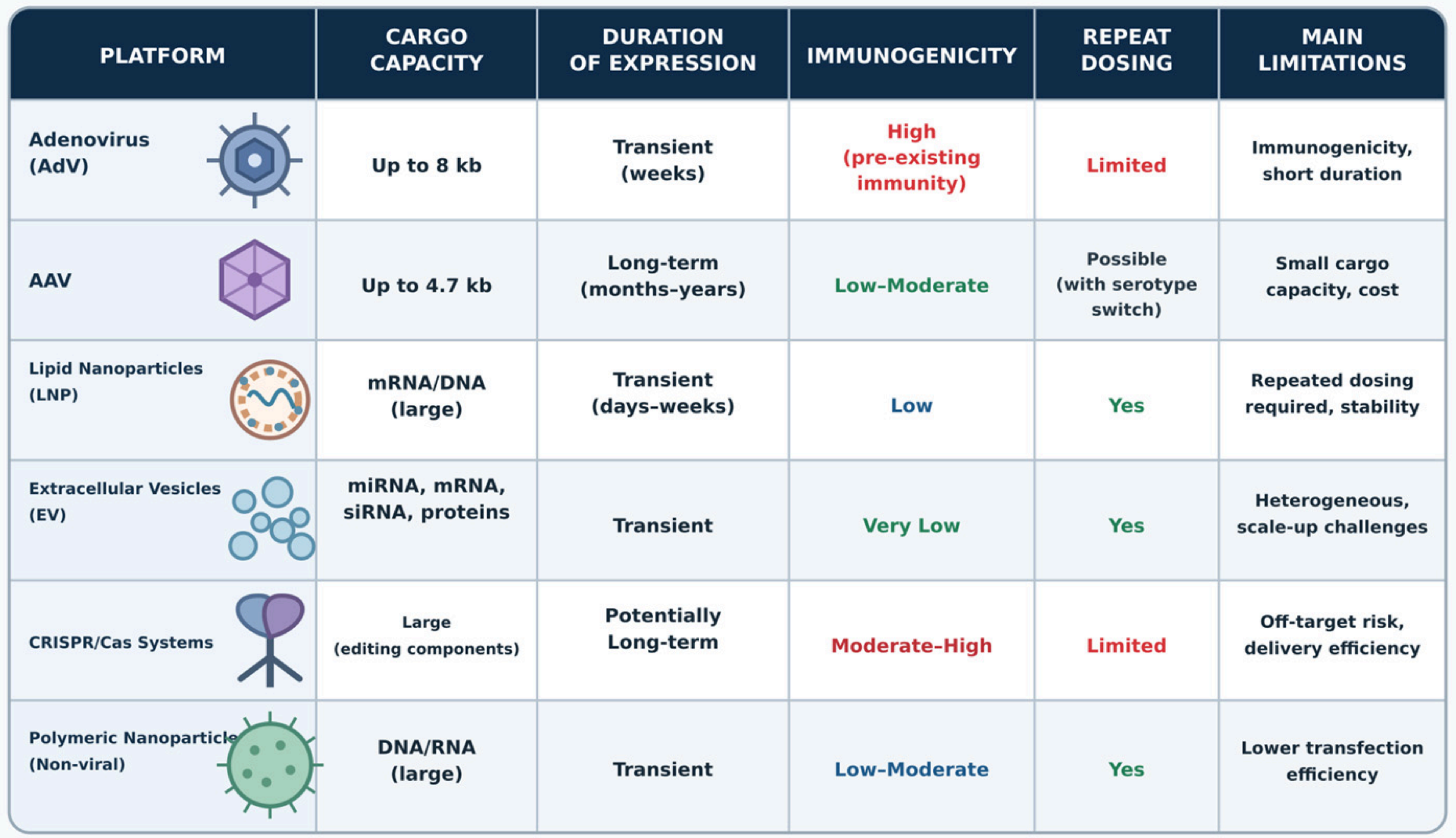
Viral and non-viral delivery systems for bronchoscopic intratumoral gene therapy in lung cancer.

The principal viral and non-viral delivery systems currently explored for intratumoral gene therapy in lung cancer are summarised in Figure 3.

### 5.2. p53 Restoration and Gene-Mediated Cytotoxic Immunotherapy: Clinical Evidence

The TP53 tumour suppressor gene is mutated or deleted in approximately 50–70% of NSCLC tumours, making its restoration a logical therapeutic target [46]. The foundational Phase I clinical trial of adenovirus-mediated wild-type p53 gene transfer in advanced NSCLC [46] established transgene expression by immunohistochemistry in post-treatment biopsies and identified a dose-dependent relationship between vector dose and p53 protein expression—the first direct evidence of pharmacodynamic target engagement by an intratumoral gene therapy agent in lung cancer.

The pivotal translational step came from Nemunaitis et al. [47], who administered adenoviral p53 in sequence with cisplatin in chemotherapy-refractory NSCLC patients. Among 25 evaluable patients, 2 achieved partial responses and 8 stable disease—an objective response rate of 8% (95% CI 1.0–26.0%, exact binomial) and disease control rate of 40% (95% CI 21.1–61.3%). These figures must be contextualised by the year 2000 treatment landscape in which they were generated and by the absence of a concurrent control arm; they nonetheless established the combinatorial principle—p53 restoration sensitising tumour cells to DNA-damaging chemotherapy—that has informed subsequent gene therapy trial design.

Gene-mediated cytotoxic immunotherapy (GMCI) represents a mechanistic evolution that converts the gene therapy needle from a corrective into an immunostimulatory instrument. GMCI delivers a herpes simplex virus thymidine kinase (HSV-TK) transgene via an adenoviral vector; systemic ganciclovir then selectively kills HSV-TK-expressing cells through toxic ganciclovir triphosphate generation, with cytotoxic metabolites transferred via gap junctions to adjacent non-transduced cells—the bystander effect—producing ICD at a scale disproportionate to the transduction efficiency of the vector. In the Phase I neoadjuvant GMCI trial in resectable NSCLC [54], post-treatment resection specimens demonstrated a median 3.2-fold increase in CD8+ TIL density compared to pre-treatment biopsies (*p* = 0.012, Wilcoxon signed-rank test), alongside PD-L1 upregulation—providing direct biological rationale for GMCI combined with checkpoint blockade in subsequent trials.

Long-term outcomes at 5 years [55] showed no late-onset vector-related adverse events, no insertional mutagenesis, and no secondary malignancies attributable to the therapeutic transgene—a durable safety record that is the most reliable finding from a non-randomised cohort. The favourable survival trends reported relative to historical controls must be interpreted with the caution warranted by non-randomised comparisons: patient selection, secular trends in supportive care, and lead-time bias from more intensive surveillance are sufficient to account for apparent survival advantages without inferring a treatment effect.

### 5.3. mRNA Constructs Targeting KRAS and EGFR: Oncogene Silencing at the Tumour Site

KRAS G12C, present in approximately 13% of all NSCLC cases and 25% of lung adenocarcinomas, and EGFR-activating mutations (exon 19 deletions and L858R), found in 10–15% of Western and 30–40% of East Asian NSCLC populations, constitute the two most clinically actionable oncogenic driver targets for intratumoral nucleic acid-based silencing [56,57]. Covalent KRAS G12C inhibitors—sotorasib and adagrasib, both FDA-approved—have transformed the systemic treatment landscape, but resistance emerges in the majority of patients within 6–12 months. Intratumoral siRNA or mRNA constructs targeting KRAS G12C degrade mutant KRAS mRNA prior to protein translation, eliminating the protein regardless of its conformational state and thereby operating through a mechanism complementary to, rather than competitive with, small-molecule inhibition.

Co-delivery of siRNA targeting both EGFR and PD-L1 via a PEGylated synthetic peptide carrier achieved 72% EGFR mRNA knockdown efficiency and 68% PD-L1 knockdown in human NSCLC cell lines, with synergistic inhibition of cell viability compared to either siRNA alone [58]. For intratumoral delivery, where the carrier does not need to survive systemic circulation, even higher knockdown efficiencies are pharmacokinetically achievable. CRISPR-Cas9 high-fidelity variants demonstrated specific disruption of the KRAS G12C allele with over 80% reduction in mutant KRAS protein expression and significant tumour volume reduction in preclinical lung cancer models [59]. The remaining translational barriers—in vivo delivery efficiency, editing specificity at therapeutic vector doses, and Cas9 immunogenicity in the human lung—are substantial but not categorically different from barriers that other CRISPR therapeutic programmes are actively addressing in the broader gene therapy literature [60,61].

### 5.4. Statistical Appraisal: Phase I Gene Therapy Evidence and the Regulatory Horizon

The statistical evidence base for intratumoral gene therapy carries a specific set of limitations that differ from those of conventional drug trials. The 3+3 dose-escalation design, derived empirically from cytotoxic chemotherapy, is not optimally suited to viral vector gene therapy: the dose-toxicity relationship for adenoviral and AAV vectors is not necessarily monotone—immune-mediated adverse events can occur at intermediate doses—and the clinically relevant toxicities (immune activation, off-target transduction, insertional mutagenesis) may manifest beyond the 28-day DLT assessment window. More adaptive designs—BOIN, mTPI, or the continual reassessment method—provide superior operating characteristics for finding the true MTD in gene therapy settings [54,55].

Vector shedding—quantified by qPCR for vector genome copies per millilitre of body fluid—is a safety endpoint unique to viral gene therapy with no analogue in conventional drug development, and its systematic reporting with confidence intervals for peak shedding concentrations and time-to-clearance estimates should be a mandatory component of Phase I reporting for endobronchial viral programmes.

The regulatory landscape has matured substantially, with 13 cell and gene therapy products approved globally in 2024—by FDA, EMA, NMPA, PMDA, and MHRA—across multiple indication areas, establishing viral vector platforms as a maturing therapeutic modality and creating regulatory precedents (RMAT designation, ATMP classification) directly applicable to intratumoral lung cancer programmes [61,62]. Jurisdictional convergence, summarised in Table 3, remains imperfect; multinational clinical trials will require deliberate alignment of data package requirements across regulatory authorities.

## 6. Mechanisms of Systemic Immune Activation

### 6.1. The Cascade from Local Injection to Systemic Immunity

When a therapeutic agent is deposited directly within a tumour, something far beyond local cell death may occur. The tumour microenvironment—long habituated to suppressing immune surveillance—can, under the right molecular conditions, become the seat of an immune response that radiates outward, reaching metastatic sites the needle never touched. The molecular cascade linking intratumoral injection to systemic immunity proceeds through five mechanistically linked steps: the induction of immunogenic cell death (ICD) and release of damage-associated molecular patterns (DAMPs); sensing of cytoplasmic DNA by cGAS and activation of STING; type I interferon production driving dendritic cell maturation; CD8+ T-cell cross-priming in tumour-draining lymph nodes; and systemic T-cell trafficking to non-injected lesions—the abscopal response. Not all deaths are immunologically equal: ICD, as defined by Galluzzi et al. in their consensus guidelines [63], is characterised by calreticulin surface exposure, extracellular HMGB1 release, and ATP secretion—a sequence that collectively converts the TME from an immunosuppressive niche into an innate immune activation zone. High local drug concentrations achievable by direct injection—but not by systemic dosing—preferentially trigger this sequence through endoplasmic reticulum stress responses.

The cGAS–STING axis, reviewed in detail by Decout et al. [64], is the innate immune system’s cytosolic surveillance circuit for double-stranded DNA. Its therapeutic activation through intratumoral STING agonist injection was demonstrated by Baird et al. [65] to produce regression of both treated and geographically remote tumours through a CD8+ T-cell-dependent mechanism—an observation that catalysed the clinical development of STING agonists including E7766 and dazostinag. Both agents have demonstrated pharmacodynamic STING target engagement in Phase I evaluation—measured by interferon-stimulated gene signature upregulation in paired biopsies [66,67]—though consistent objective responses at distant sites have remained elusive, reflecting an inherent biological tension: chronic low-grade STING signalling paradoxically drives PD-L1 upregulation, T-cell exhaustion, and potential metastatic promotion [68,69]. The therapeutic window is narrow, and its boundaries in human lung cancer remain to be precisely defined.

### 6.2. Polyclonal T-Cell Priming, Epitope Spreading, and TLS Formation

One of the most immunologically compelling features of intratumoral therapy—and one that distinguishes it categorically from single-antigen vaccination—is its capacity to trigger polyclonal T-cell responses against the full breadth of a patient’s tumour neoantigen repertoire. When intratumoral injection induces ICD, the entire antigenic inventory of the dying tumour is simultaneously released into an activated, adjuvant-rich microenvironment and cross-presented as multiple distinct peptide-MHC complexes to the naïve T-cell repertoire [63,70]. Epitope spreading—the de novo expansion of T-cell clonotypes targeting antigens not present in the injected lesion—has been documented in patients receiving intratumoral therapies with checkpoint blockade, providing in vivo evidence that locoregional treatment can reprogram systemic immunity at a scale unachievable by systemic monotherapy. Even if immune escape through antigen loss occurs at one neoepitope, parallel T-cell clones targeting alternative tumour antigens may maintain systemic tumour control—a redundancy absent from single-epitope therapeutic strategies.

Tertiary lymphoid structures (TLS)—ectopic lymphoid aggregates forming within the TME under conditions of chronic immune stimulation—represent the organisational infrastructure that sustains polyclonal immune responses locally [71,72,73]. Their prognostic and predictive value in NSCLC has been quantitatively established by two independent meta-analyses. Liu et al. [74] synthesised 17 studies encompassing 4291 patients and demonstrated TLS presence associated with improved OS (pooled HR 0.59, 95% CI 0.48–0.72, *p* < 0.001) and PFS (HR 0.61, 95% CI 0.50–0.74), with low-to-moderate between-study heterogeneity (I2 = 34% and 28%, respectively)—consistent with a biologically reproducible effect. Li et al. [75] confirmed TLS positivity predicted objective response to ICI (OR 2.84, 95% CI 1.73–4.65) and OS benefit (HR 0.55, 95% CI 0.44–0.69), though higher heterogeneity (I2 = 52%) reflected variability in TLS scoring methods across studies.

A critical statistical distinction separates TLS as a prognostic from a predictive biomarker. A biomarker is prognostic if it predicts patient outcome regardless of treatment; it is predictive if it differentially identifies patients who benefit from a specific intervention. The statistical test for predictive value—a treatment-by-biomarker interaction term in a Cox regression model—requires randomised data and is rarely reported in observational TLS studies. Most current evidence demonstrates that TLS-positive tumours have better outcomes (a prognostic signal) without formally establishing whether TLS specifically predicts differential benefit from intratumoral therapy. Designing trials that enrol patients based on a prognostic rather than a predictive biomarker risks enriching a generally good-prognosis population rather than identifying the patients most likely to respond to the specific intervention under investigation.

### 6.3. Cold-to-Hot Tumour Conversion: Phenotypic Barriers and Combined Targeting

**Figure 4:**
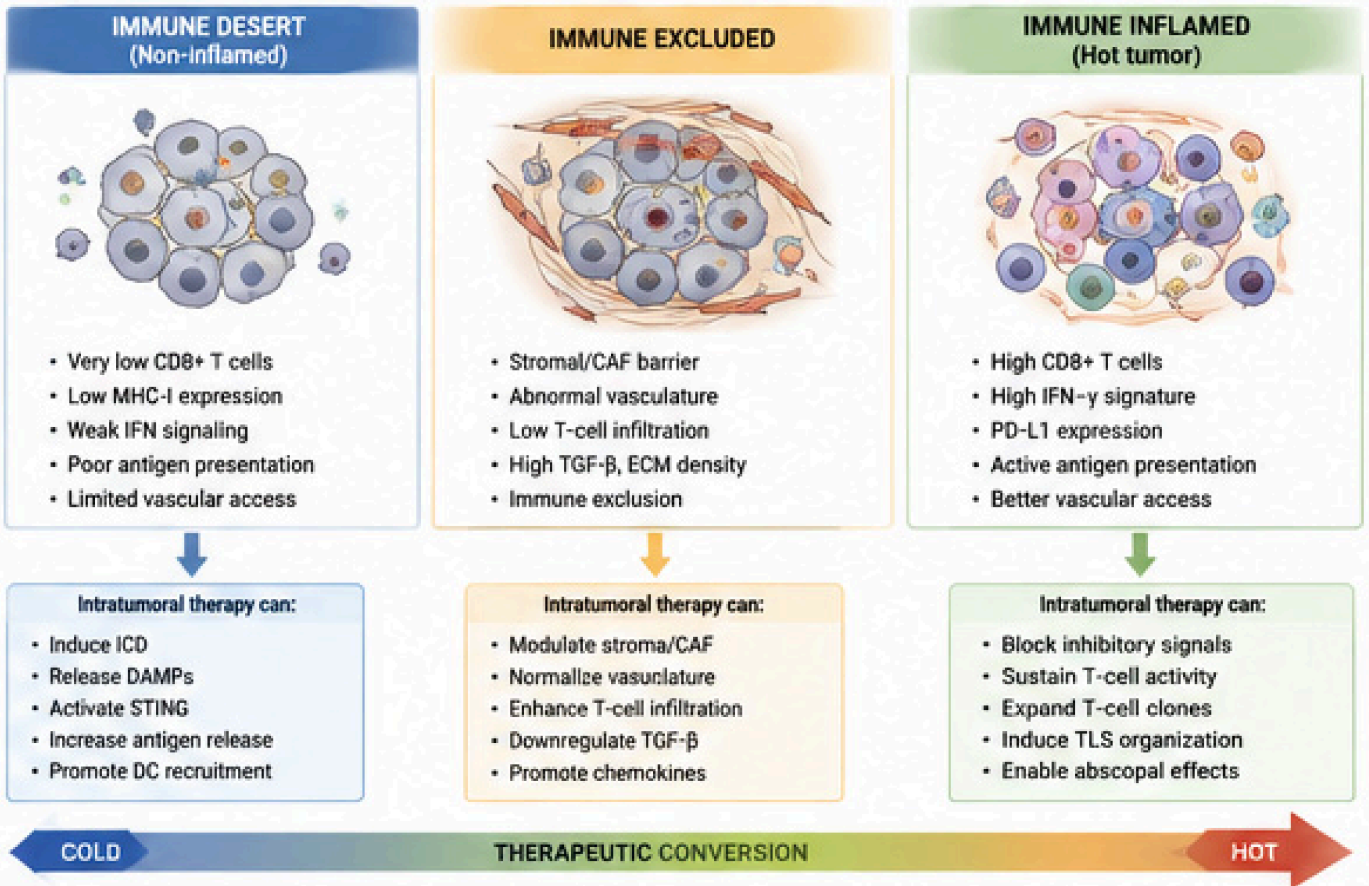
Mechanisms of cold-to-hot tumour conversion in NSCLC following intratumoral immunotherapy.

Approximately 70% of advanced NSCLC tumours are immunologically cold—characterised by absent or sparse T-cell infiltration, low MHC class I expression, and a myeloid-dominant TME that renders them refractory to systemic checkpoint blockade [9,76]. Three mechanistically distinct phenotypes of T-cell exclusion have been characterised: the immune desert (T cells absent from both core and invasive margin), the immune excluded tumour (T cells trapped at the tumour periphery by stromal barriers or aberrant vasculature), and the immune inflamed tumour (T cells present but functionally suppressed by checkpoint ligands or metabolic competition) [8,76]. The principal phenotypic barriers underlying immune exclusion and the mechanisms through which intratumoral therapies may promote cold-to-hot tumour conversion are summarised in Figure 4.

Intratumoral delivery addresses all three: ICD induction generates de novo inflammation in the desert phenotype; STING agonism and cytokine delivery can disrupt stromal barriers in excluded tumours; local checkpoint injection addresses suppressive signalling within the inflamed TME [76]. Systematic cataloguing of cold-to-hot conversion mechanisms in NSCLC [76] has identified cancer-associated fibroblast reprogramming, MDSC depletion, and NK cell recruitment as the most clinically tractable targets—each actionable through components of the intratumoral therapeutic toolkit reviewed in Section 3 and Section 4. Durable cold-to-hot conversion likely requires simultaneous targeting of at least two non-overlapping immunosuppressive mechanisms, providing the mechanistic rationale for combination intratumoral regimens rather than single-agent approaches.

### 6.4. Statistical Appraisal: Translational Validity, Publication Bias, and Inference Limits

The mechanistic literature on cGAS–STING, ICD, and the abscopal effect is architecturally impressive but quantitatively fragile at the base. The overwhelming majority of STING agonist and abscopal effect studies derive from murine syngeneic models characterised by high immunogenicity and subcutaneous implantation—conditions that systematically overestimate anti-tumour immune responsiveness relative to spontaneous human lung cancers [68]. Median sample sizes per experimental group of 5–10 animals provide adequate power only to detect standardised effect sizes above 1.0; effects of this magnitude are rarely reproduced at an equivalent scale in human trials. Effect sizes in most preclinical publications are represented graphically without numerical estimates or confidence intervals, precluding formal power assessment.

The TLS meta-analytical literature suffers from an additional inferential limitation: neither the Liu et al. [74] nor the Li et al. [75] meta-analysis reports Egger’s test statistics or funnel plot symmetry assessments for publication bias. Given that TLS studies reporting null associations are systematically underrepresented in the literature searches, the pooled HRs should be regarded as upper bounds of the true effect size rather than unbiased estimates. The hazard ratio itself carries a further limitation: the proportional hazards assumption is frequently violated in immunotherapy trials, where survival curves may cross or converge as durable responders separate from non-responders over time.

Reporting restricted mean survival time (RMST) alongside HRs and formal testing of the proportional hazards assumption using Schoenfeld residuals would substantially improve the interpretability of survival analyses in this field. Additionally, advanced NSCLC patients face meaningful competing risks from non-cancer mortality; cause-specific and subdistribution hazard models (Fine-Gray) provide more appropriate estimation frameworks than standard Kaplan–Meier analyses when non-cancer death is a clinically significant competing event.

## 7. Predictive Biomarkers of Systemic Response to Intratumoral Therapy

### 7.1. Prognostic vs. Predictive: The Foundational Statistical Distinction

The search for biomarkers in oncology immunotherapy has generated a literature of considerable volume and inconsistent rigour. The prognostic–predictive distinction, introduced in Section 5.2 in the context of TLS, applies equally to every candidate biomarker reviewed here. A biomarker’s association with clinical outcome in observational data establishes its prognostic value; the formal test of predictive value—a statistically significant treatment-by-biomarker interaction term in a randomised trial—remains rare across the intratumoral therapy biomarker literature [77]. The clinical utility of a prognostic biomarker, however statistically robust, is limited if it does not differentially predict treatment benefit. Quantitative performance characteristics of the five principal candidate biomarkers—TMB, PD-L1, CD8+ TILs, TLS, and ctDNA—are summarised in Table 4, which provides AUC estimates, sensitivity and specificity at clinical thresholds, OS hazard ratios, and information on threshold derivation methodology.

**Figure 5:**
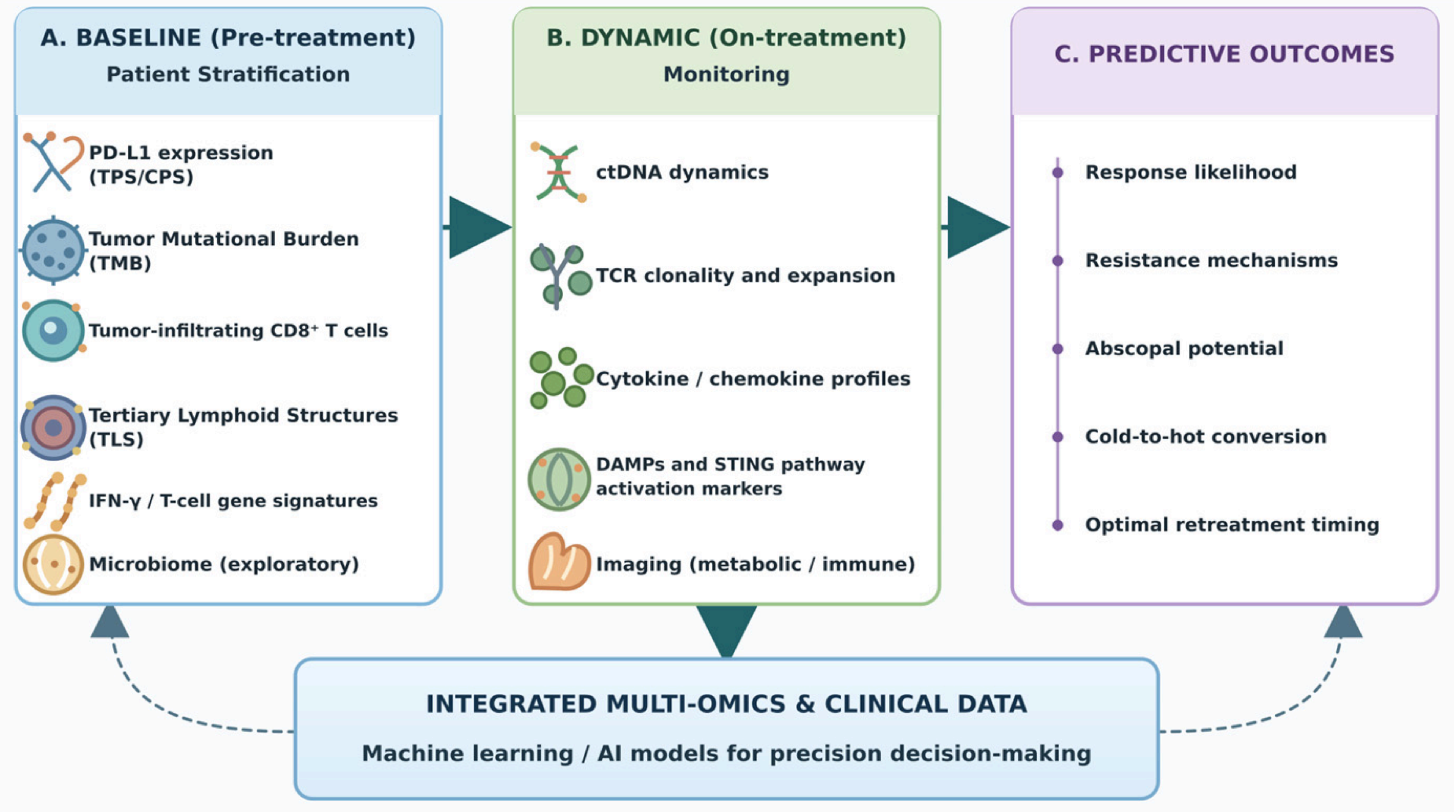
Biomarker framework for prediction and monitoring of systemic response to intratumoral immunotherapy in NSCLC.

The conceptual integration of baseline, dynamic, and composite biomarkers relevant to intratumoral immunotherapy response is illustrated in Figure 5.

### 7.2. Tumour Mutational Burden: Threshold Derivation and Discriminatory Limits

The FDA’s 2020 tumour-agnostic approval of pembrolizumab for TMB-high solid tumours (≥10 mutations per megabase, Foundation Medicine CDx assay) established the first regulatory threshold for TMB-based patient selection. The 10 mut/Mb cutoff was selected post hoc as the Youden Index-maximising point on the TMB-ORR ROC curve in the KEYNOTE-158 cohort—an optimisation on a dataset not prespecified for this purpose [82,83]. The AUC of this ROC curve for predicting objective response in the NSCLC cohort was approximately 0.64–0.68: statistically superior to chance but reflecting only modest discriminatory ability. At 10 mut/Mb, sensitivity for detecting responders is approximately 57–65% and specificity 63–70%—meaning a substantial fraction of patients who would respond to pembrolizumab are excluded by this criterion, and a comparable fraction of non-responders are classified as eligible.

Three further limitations constrain TMB’s clinical utility. First, binarising a continuous distribution discards prognostic information: analyses treating TMB as a continuous predictor in proportional hazards regression consistently show a more informative dose–response relationship than binary categorisation. Second, inter-platform variability in TMB quantification is substantial—whole-exome sequencing versus targeted panel sequencing can generate estimates differing by 2–3 mut/Mb for the same tumour, creating classification uncertainty around the regulatory threshold [82]. Third, synonymous mutations—which contribute to raw TMB counts but cannot generate neoantigens—introduce a biological imprecision built into the very definition of the biomarker. Effective TMB (mutations generating verifiable neoantigens) may be a superior predictive metric, though its standardisation across platforms remains incomplete.

### 7.3. PD-L1, CD8^+^ TILs, and Composite Immune Biomarkers

PD-L1 expression as a tumour proportion score (TPS) remains the most widely used clinical biomarker for ICI eligibility in NSCLC. Four companion diagnostic antibody clones are in active clinical use (22C3, 28–8, SP142, SP263), with concordance between the 22C3 and 28–8 clones quantified by kappa statistics at approximately κ = 0.60–0.72 at the ≥50% TPS threshold—substantial but imperfect agreement, meaning that the classification of an individual patient as PD-L1 high or low can differ between assays [82]. Intratumoural spatial heterogeneity further complicates single-biopsy PD-L1 assessment: expression varies substantially across different regions of the same tumour, between primary and metastatic sites, and across treatment timepoints. The threshold multiplicity problem—different clinical approvals using different PD-L1 cutoffs (≥1%, ≥25%, ≥50%) derived from different trials, platforms, and populations—makes cross-trial comparison of PD-L1-stratified analyses methodologically unreliable.

Platelet PD-L1, measured in circulating platelets that sample the intratumoural vascular environment and absorb surface proteins from tumour cells, offers a spatially integrated measure of tumour PD-L1 exposure that single-site biopsy cannot provide—with a reported AUC of 0.72 (95% CI 0.63–0.81) for predicting ICI response in NSCLC [84]. CD8+ TIL density, while prognostically informative (pooled HR for OS approximately 0.61, 95% CI 0.48–0.77) [79], has not been formally tested as a predictive biomarker for intratumoral therapy. Composite biomarker classifiers combining PD-L1 TPS with CD8+ TIL density improve predictive performance over either marker alone in a systematic review, supporting the development of multivariate biomarker strategies for intratumoral therapy patient selection [78].

### 7.4. Circulating Tumour DNA: The Kinetic Response Signal

ctDNA is unique among the candidate biomarkers reviewed here in its temporal dimension: unlike TMB, PD-L1, and TIL density—static measurements reflecting tumour biology at a single timepoint—ctDNA provides a dynamic signal that changes in response to treatment, offering near real-time monitoring of the systemic immune response to local intratumoral intervention [85]. Methylation-based ctDNA serial monitoring in a longitudinal NSCLC cohort demonstrated that a ≥50% decrease in ctDNA methylation signal at 6 weeks from pembrolizumab initiation predicted objective response with sensitivity 72–78% and specificity 78–83% [80]. Concordance between the ctDNA-defined response and the RECIST 1.1 imaging response was quantified at Cohen’s κ = 0.64—substantial agreement, with discordant cases predominantly reflecting the pseudoprogression phenomenon.

Longitudinal tracking of ctDNA alongside circulating immune cell compartments—CD4+ and CD8+ T-cell subsets, NK cells, monocytes—identified patients achieving molecular remission (ctDNA clearance with expanding tumour-reactive T-cell clones) versus those with discordant kinetics [81]. For intratumoral therapy, where the therapeutic goal is specifically the induction of systemic immune activation, this multi-parameter ctDNA/immune tracking approach represents the most mechanistically aligned monitoring strategy available. Pre-specification of ctDNA response endpoints—including the analytical threshold for meaningful change, the timepoint for assessment, and the classification of discordant kinetics—should be a mandatory component of intratumoral therapy trial statistical analysis plans, alongside iRECIST as the primary imaging assessment framework.

### 7.5. The Clinical Utility Threshold and the Pre-Specification Imperative

The biomarker literature reviewed here illustrates a persistent tension between statistical association and clinical utility. A biomarker with AUC 0.67—approximately the discriminatory performance of TMB at its regulatory threshold—selects patients with a positive predictive value of approximately 31–35% and negative predictive value of 87–90%, given an underlying response rate of ~20% in unselected NSCLC. Whether this level of discrimination justifies withholding treatment from biomarker-negative patients depends on the relative harm of over- and under-treatment—a clinical value judgement that statistical performance metrics alone cannot resolve [77]. Quantifying the number needed to test (NNT-to-benefit) alongside the number incorrectly classified (NNM) provides a more decision-relevant framing than AUC alone and should accompany biomarker performance reporting in intratumoral therapy trials.

The pre-specification imperative is absolute. The history of oncology biomarker development is marked by post hoc thresholds—derived from and tested in the same dataset—that proved non-replicable in independent cohorts and nonetheless informed clinical decisions for thousands of patients before their limitations were recognised. Pre-registration of biomarker hypotheses, prospective split-sample or cross-validation designs for threshold discovery, mandatory prospective replication in an independent cohort before clinical adoption, and FDR correction for multiple comparisons in exploratory analyses are methodological standards that the intratumoral therapy field must adopt from the outset rather than retrofit after the fact [82,83].

## 8. Clinical Trial Data and Safety Profiles of Endobronchial Intratumoral Therapies

### 8.1. Trial Landscape and Safety

The clinical trial landscape for endobronchial intratumoral therapy is, as of this writing, composed almost exclusively of Phase I dose-escalation studies, small prospective cohorts, and Phase I/II expansion cohorts—study designs powered to identify dose-limiting toxicities, not to establish efficacy. Thirteen trials and prospective cohort studies spanning EBUS-guided intratumoral chemotherapy, viral gene therapy, dendritic cell vaccination, mRNA-LNP platforms, oncolytic virotherapy, STING agonists, and non-viral nanoparticle delivery are summarised in Table 5. Not a single randomised controlled trial of endobronchial intratumoral therapy against a standard-of-care comparator has been completed in NSCLC. The entire body of efficacy evidence is therefore single-arm, subject to the selection biases and absence of counterfactual that single-arm designs cannot overcome regardless of their statistical execution [11,21].

The most clinically actionable conclusion from the available data is also the most consistent: endobronchial intratumoral delivery is safe. Across all 13 trials, procedure-related serious adverse events—pneumothorax, haemoptysis, or airway perforation attributable to the delivery procedure itself—have not been reported at frequencies exceeding those of diagnostic bronchoscopy (serious complication rate approximately 0.5–1.5% of procedures) [14,19,86]. Drug-specific adverse events differ by category. For intratumoral chemotherapy (cisplatin, paclitaxel), the dominant signal is injection-site inflammation and transient fever without the haematological, renal, or neurological toxicities that characterise intravenous administration. For viral vectors, dose-limiting toxicities have not been observed at the dose levels evaluated in published reports [54,55]. For mRNA-LNP and STING agonist platforms, cytokine release syndrome has occurred only at grades 1–2 without grade 3 or 4 events at reported dose levels [32,66,67]. The longest available safety follow-up—60 months from the GMCI programme—shows no late-onset vector-related adverse events, insertional mutagenesis events, or secondary malignancies [55]. This reassuring long-term record must be contextualised: with cohort sizes of 15–30 patients, statistical power to detect rare late adverse events (occurring in 1 in 500 patients) is essentially nil.

### 8.2. Efficacy Signals: Biological Activity Without Definitive Proof

Across categories, intratumoral delivery consistently modifies the tumour immune microenvironment in the predicted direction: CD8+ TIL density increases, PD-L1 is upregulated as an adaptive immune resistance response, IFN-γ is detected in post-treatment biopsies, and pharmacodynamic markers of STING pathway activation confirm that delivered agents are reaching their molecular targets [32,44,54,66,87]. These biological signals establish that the delivery works and that the immune consequences are in the right direction—but they do not answer the clinical question: does this translate into improved survival outcomes?

The most mature efficacy data come from the p53/GMCI programme and the neoantigen-targeted DC vaccination cohort. Nemunaitis et al. [47] reported ORR 8% (95% CI 1.0–26.0%) and DCR 40% (95% CI 21.1–61.3%) with adenoviral p53 plus cisplatin in chemotherapy-refractory NSCLC—historically meaningful in their era but providing a floor rather than a ceiling for contemporary intratumoral combination therapy. The GMCI 5-year data [55] and the neoantigen DC vaccination cohort reporting a median PFS of 8.6 months [43] represent promising directional signals from non-randomised cohorts. Comparisons of these figures against historical benchmarks or contemporary single-arm immunotherapy trials are subject to the full array of confounders that make uncontrolled comparisons unreliable—patient selection, secular trends in supportive care, and lead-time bias from more intensive surveillance during trial participation.

### 8.3. Patient Selection Heterogeneity and the Evidence Hierarchy

Eligibility criteria across the 13 trials in Table 5 vary substantially: stage ranges from resectable Stage I (GMCI neoadjuvant cohort) to extensive-stage SCLC (RT-01); prior treatment requirements range from treatment-naïve to multiply pretreated; and technical eligibility—requiring peribronchial tumour location, a CT bronchus sign for ENB access, or EBUS-visualisable target—introduces a patient subgroup with favourable anatomical features that may differ systematically from the broader NSCLC population in ways not captured by standard prognostic variables [21]. Cross-study comparison of outcome metrics is therefore methodologically unreliable, and any informal pooling of response rates or survival figures from this evidence base would risk producing a spuriously precise estimate of a biologically meaningless average.

The statistical evidence hierarchy for endobronchial intratumoral therapy sits at Level III–IV on the Oxford CEBM scale. What would advance this to Level I–II? A two-arm randomised Phase II trial with progression-free survival as the primary endpoint—assuming a median PFS of 4 months in the control arm, a target HR of 0.65, one-sided α = 0.10, and 80% power—would require approximately 160 events and 200–220 patients [11,21]. This sample size is achievable within a multicentre consortium over 3–4 years. The scientific machinery for this trial exists; the institutional and financial commitment to build the required consortium has not yet materialised.

## 9. Combining Intratumoral Therapy with Stereotactic Body Radiotherapy

### 9.1. Radiobiological Synergy and the Immunostimulatory Dose Window

**Figure 6:**
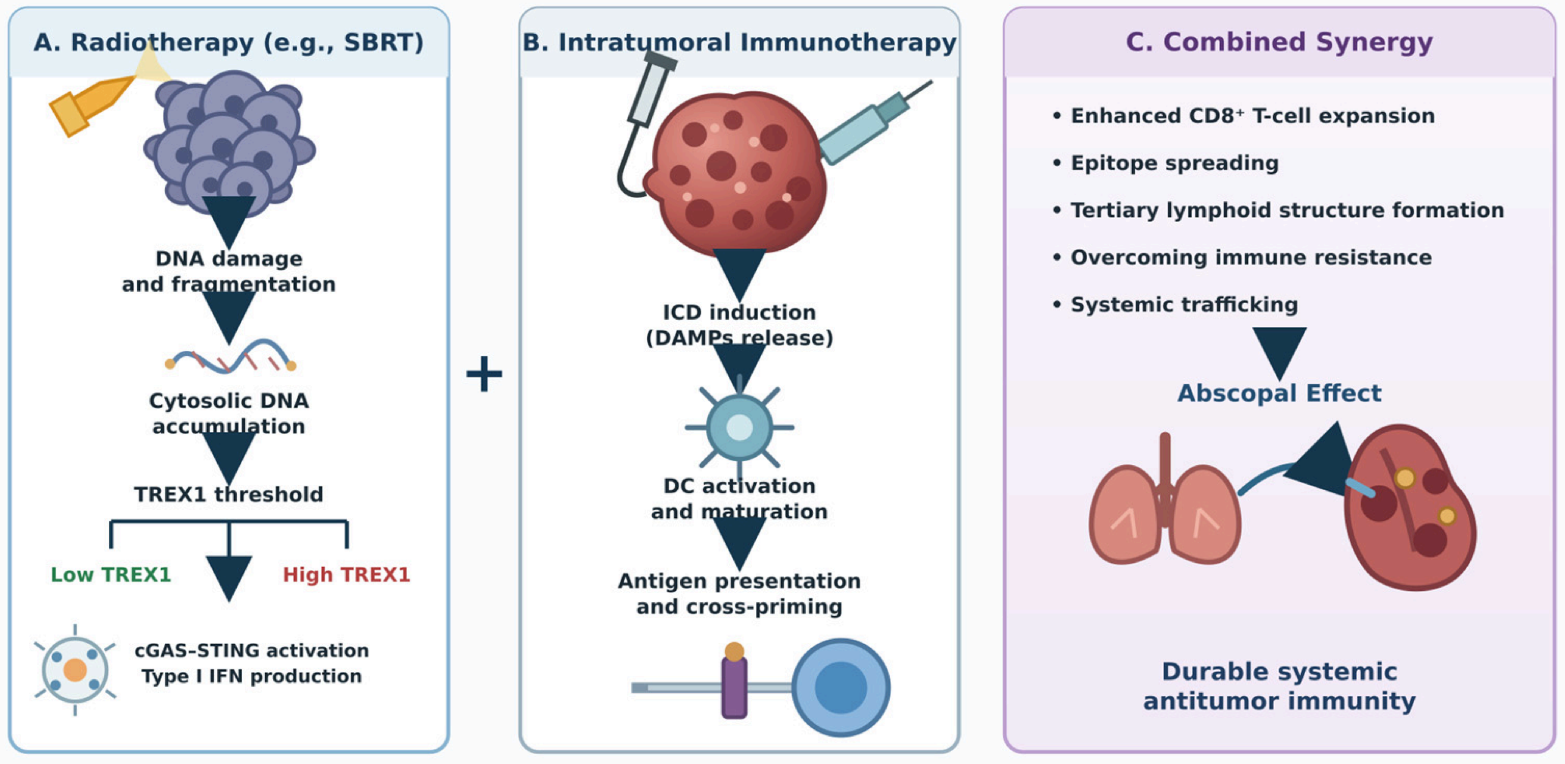
Mechanistic synergy between stereotactic radiotherapy and intratumoral immunotherapy in lung cancer.

Radiation and intratumoral immunotherapy are mechanistically complementary partners [70,89]. Conventional fractionated radiotherapy (1.8–2 Gy per fraction) produces predominantly tolerogenic cell death; stereotactic body radiotherapy (SBRT), delivering ablative doses of 8–20 Gy per fraction over 3–5 fractions, generates irreparable DNA damage, induces mitotic catastrophe, and produces sufficient cytoplasmic double-stranded DNA to activate the cGAS–STING pathway in both tumour cells and infiltrating innate immune cells [67,69]. This creates an immunostimulatory microenvironment—characterised by DAMP release, dendritic cell activation, and type I interferon production—that amplifies the cascade initiated by intratumoral injection. The biological effective dose (BED) at 8–10 Gy per fraction ranges from 54 to 90 Gy for tumour tissue (α/β = 10 Gy), representing the immunostimulatory window for SBRT. Critically, very high single fractions (>12–15 Gy) trigger upregulation of TREX1—a cytoplasmic DNase that degrades cytosolic double-stranded DNA before cGAS can sense it—suppressing the STING pathway and reversing the immunostimulatory advantage. This dose dependency defines an upper boundary for the productive RT–IT interaction [67]. The mechanistic interaction between stereotactic radiotherapy and intratumoral immunotherapy is summarised in Figure 6.

CD73, an ectoenzyme that degrades extracellular ATP into immunosuppressive adenosine, represents a critical molecular brake on the RT–IT abscopal cascade. An et al. [90] demonstrated in preclinical NSCLC models that CD73 blockade combined with radiotherapy potentiated systemic anti-tumour immunity through a STING-dependent mechanism, significantly increasing the CD8+ T-cell to regulatory T-cell ratio at non-irradiated tumour sites, with formally demonstrated synergism (combination index < 1.0). This mechanistic finding positions CD73 inhibition as a rational addition to RT–intratumoral combination regimens.

### 9.2. Clinical Evidence and Statistical Appraisal

Huang et al. [91] evaluated pembrolizumab with hypofractionated radiotherapy in patients with advanced, immunologically cold NSCLC. TCR clonotype diversity—measured by Shannon entropy from peripheral blood TCR sequencing—increased significantly from baseline in patients receiving concurrent pembrolizumab and radiation (mean ΔH = 0.38 bits, 95% CI 0.21–0.55, *p* < 0.001), correlating with partial or complete responses at non-irradiated lesions (Spearman r = 0.57, *p* = 0.003). Formal interaction testing confirmed that TCR diversification was attributable to the radiation component, not immunotherapy alone—establishing mechanistic evidence for genuine RT–ICI synergy rather than additive effects.

The pooled analysis by Wang et al. [92]—incorporating randomised and observational data on immunotherapy with or without SBRT in advanced lung cancer—reported a pooled HR for OS of 0.71 (95% CI 0.58–0.87) favouring the combination, with I2 = 38% reflecting low-to-moderate between-study heterogeneity. Three statistical limitations constrain interpretation. First, contributing studies were not designed with the SBRT–immunotherapy combination as the primary hypothesis, creating a quasi-randomised exposure susceptible to confounding by indication. Second, SBRT timing (concurrent vs. sequential) varied substantially across studies, and the interaction between timing and outcome was not formally tested. Third, SBRT BED at the tumour ranged from 54 to 151 Gy—a range spanning both the immunostimulatory and TREX1-mediated suppression zones, potentially diluting the pooled treatment effect [92,93].

The rational integration of SBRT with endobronchial intratumoral delivery raises a sequencing question that current data cannot resolve. Arguments for concurrent delivery—radiation-induced DAMP release potentiating intratumoral agent uptake—are countered by the practical observation that post-RT oedema and neutrophil infiltration may impair drug distribution and bronchoscopic access. Sequential delivery (SBRT first, intratumoral therapy 5–10 days later, once acute inflammation resolves) is a testable hypothesis that should be pre-specified as a stratification variable in any prospective RT–IT combination trial, with pre-specification of the SBRT BED range to restrict dosimetry to the immunostimulatory window (54–90 Gy) [70,91].

## 10. Global Feasibility, Regulatory Landscapes, and Socioeconomic Barriers

### 10.1. Infrastructure Requirements and Geographic Maldistribution

**Figure 7:**
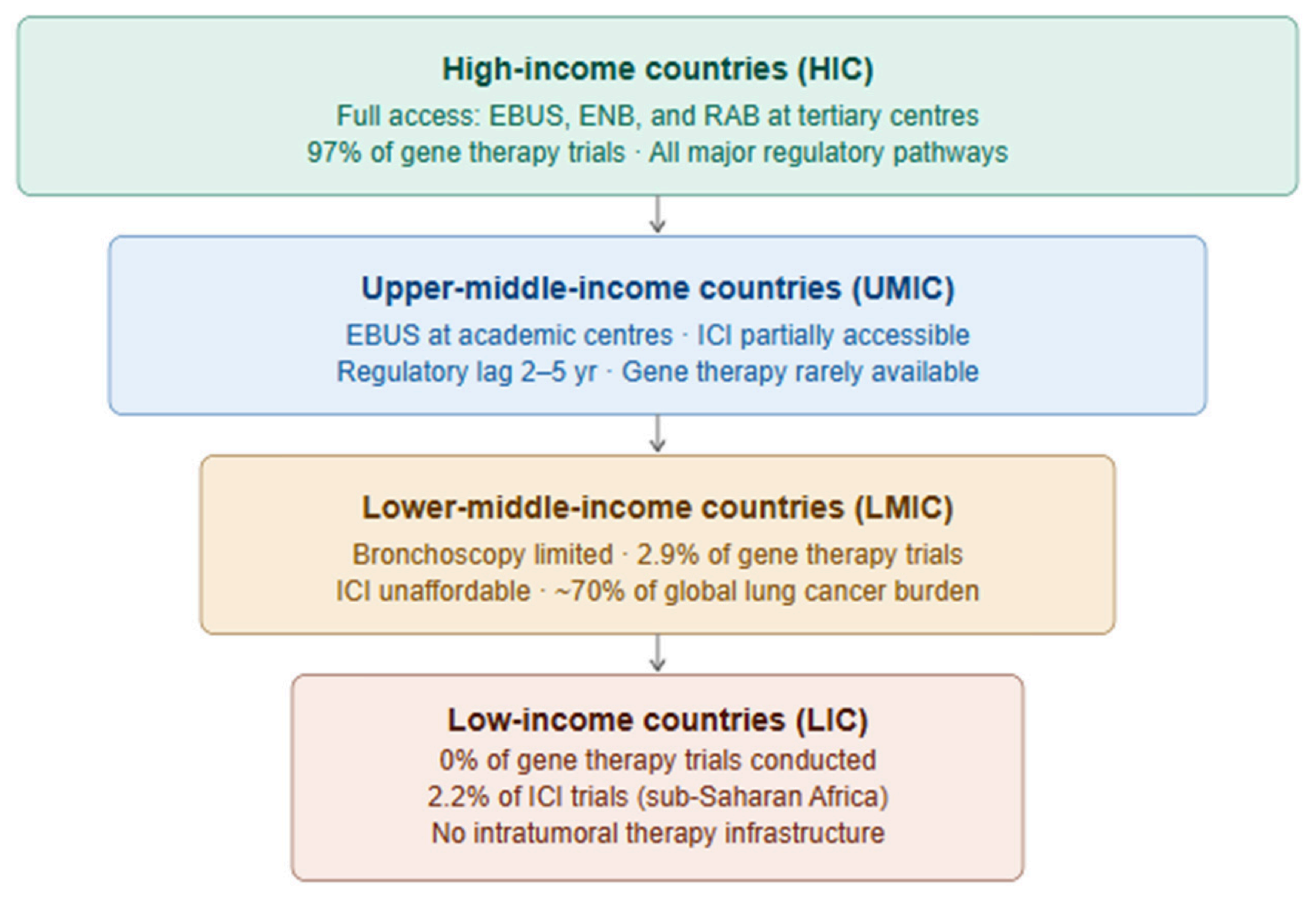
Tiered feasibility of endobronchial intratumoral therapy across country income groups.

Endobronchial intratumoral therapy requires, at minimum, a bronchoscopy suite with EBUS capability, a trained interventional pulmonologist with competency in therapeutic transbronchial injection, anaesthesia support, aseptic pharmacy preparation for intratumoral drug formulations, and a multidisciplinary oncological team structure. For viral vector gene therapy or mRNA-LNP platforms, additional requirements—biosafety level II containment, cold chain storage, and real-time imaging guidance—substantially increase the infrastructure threshold [10,11,21]. The tiered structure of global access to this infrastructure is illustrated in Figure 7. In high-income countries (HICs), EBUS bronchoscopy is available at most tertiary oncology centres and a growing proportion of secondary hospitals. In low- and middle-income countries (LMICs), which bear approximately 70% of the global lung cancer burden, access remains concentrated in a small number of metropolitan academic centres—with vast geographic regions having no EBUS access whatsoever. Robotic bronchoscopy platforms, costing in excess of USD 750,000 per system, are effectively inaccessible to healthcare systems operating on budgets below USD 100 per capita annually.

The geographic maldistribution of clinical trials quantifies this inequity in numbers that are difficult to dispute. Doxzen et al. [94] found that zero percent of gene therapy trials had been conducted in low-income countries and only 2.9% in middle-income countries—with 97.1% conducted exclusively in high-income settings. The SITC Global Access Committee [95] documented that ICI trials in sub-Saharan Africa accounted for only 2.2% of global ICI trial enrolment despite the region representing approximately 16% of the global population. Eldridge et al. [96] surveyed 223 oncology clinicians practising in LMICs: inadequate funding was identified as the primary barrier by 87% of respondents, insufficient trial infrastructure by 73%, regulatory complexity by 68%, and shortage of trained clinical research personnel by 61%. These barriers form an interlocking system—the absence of any single component is sufficient to prevent trial participation—and addressing them requires coordinated investment across all four domains simultaneously.

### 10.2. Economic Barriers and Cost-Effectiveness Considerations

Cell and gene therapies approved in 2024 carry list prices ranging from approximately USD 300,000 to USD 3,500,000 per treatment course [62]. A therapy costing USD 1,000,000 that generates 10 additional quality-adjusted life-years (QALYs) produces a cost per QALY of USD 100,000—within the WHO-recommended cost-effectiveness threshold of 1–3 times GDP per capita for HICs with GDP per capita above USD 50,000, but 100-fold above the equivalent threshold for a country with GDP per capita of USD 1000. Financial modelling by Tay-Teo et al. [97] demonstrated that even modest ICI monotherapy regimens—substantially cheaper than gene therapy—were not cost-effective in low-income countries, with incremental cost-effectiveness ratios exceeding USD 500,000 per QALY. Sensitivity analyses identified drug price as the parameter most influential on cost-effectiveness (a 10% price reduction improving the ICER by 8–12%), followed by uncertainty in the PFS hazard ratio (±20% uncertainty shifting the ICER by 15–22%). For endobronchial intratumoral delivery, which adds procedure costs of USD 2000–8000 per session on top of drug costs, the economic case in LMIC settings is even more challenging absent substantial price reductions or health system subsidisation.

Low-dose immunotherapy—reviewed by Hoyek et al. [98]—achieves anti-tumour activity at 20–30% of the standard ICI dose, with substantially reduced cost, in a proportion of patients. Whether this dose-reduction principle translates to intratumoral gene therapy or viral vector platforms—where dose is defined by vector genome copies—requires specific pharmacodynamic investigation. The regulatory landscape for gene and cell therapy, condensed by jurisdiction in Table 2, shows accelerated assessment pathways (RMAT, ATMP, Sakigake) that are directly applicable to intratumoral lung cancer programmes. Regulatory convergence across jurisdictions—currently imperfect, actively pursued through ICMRA and ICH S12—will be a critical enabler of the multinational trials required to generate generalizable evidence, but its benefits will accrue primarily to middle-income countries with established regulatory agencies, not to the lowest-income settings where regulatory capacity itself is the primary limiting factor [61,62].

### 10.3. Strategies for Expanding Access and the Global Equity Imperative

Two structural models offer partial solutions to the access gap. The Global Gene Therapy Initiative (GGTI) [99] has piloted a hub-and-spoke model equipping LMIC academic centres with infrastructure, trained personnel, and regulatory support through philanthropic funding and academic collaboration—with the Uganda and India pilot programmes requiring approximately USD 2.8 million in direct infrastructure costs per site to achieve gene therapy trial readiness. A tiered feasibility framework proposed by Firdaus et al. [21] stratifies intratumoral interventions by infrastructure requirements: Tier 1 (EBUS-guided IT chemotherapy—accessible to secondary hospitals in middle-income countries using existing endoscopy infrastructure); Tier 2 (mRNA-LNP and oncolytic virotherapy—requiring targeted pharmacy investment and EBUS/ENB platforms); and Tier 3 (viral vector programmes—requiring dedicated gene therapy infrastructure). This tiered model provides a roadmap for phased capacity-building that avoids the false choice between waiting for universal access and treating only the wealthiest populations.

The equity dimension of this review is not peripheral to its scientific content—it is central to it. A therapeutic strategy mechanistically sound and clinically actionable in twenty countries while structurally inaccessible to the remaining one hundred and sixty is not, in any meaningful sense, a solution to the global lung cancer burden. The scientific community’s obligation extends beyond demonstrating that intratumoral delivery works: it includes the obligation to build the evidence base, the infrastructure, and the policy frameworks required to extend that access as broadly as the biology allows.

## 11. Future Directions: The Bronchoscope as a Platform for Precision Immuno-Oncology

### 11.1. AI-Guided Navigation and the Single-Session Paradigm

The integration of artificial intelligence into bronchoscopic platforms is progressing from the diagnostic domain, including convolutional neural network-based lesion classification and automated lymph node measurement from EBUS images, toward a more complex therapeutic role in AI-guided intratumoral intervention. In this setting, AI should not be understood merely as a navigation aid but as a decision-support layer that may influence target selection, injection planning, dose distribution, intraprocedural adaptation, and post-treatment response assessment. This broader role is particularly relevant for intratumoral therapy because the therapeutic effect depends not only on reaching the lesion but also on selecting the biologically most informative and therapeutically actionable region within a heterogeneous tumour [23,100].

AI-guided target selection could integrate preprocedural CT, PET/CT, radiomic tumour phenotypes, bronchoscopic accessibility, lesion vascularity, necrotic fraction, proximity to major vessels or airways, and baseline molecular or immune biomarkers to prioritise which lesion, or which region within a lesion, should be injected. For example, a centrally accessible lesion with high metabolic activity, viable peripheral tumour rim, low necrotic burden, and radiological features suggesting immune exclusion may be a more rational target than a largely necrotic lesion with poor expected drug retention. In multifocal disease, AI-based lesion prioritisation could support selection between a primary tumour, mediastinal nodal disease, and metastatic pulmonary deposits by estimating procedural feasibility, expected drug dispersion, and likelihood of systemic immune priming.

Beyond lesion selection, AI may guide the technical development of local delivery platforms. Three-dimensional tumour reconstruction from CT or cone-beam CT could be combined with real-time EBUS or robotic bronchoscopy data to simulate injection trajectories, define the number and spatial arrangement of injection points, and estimate the fraction of tumour volume exposed to therapeutic concentrations. Pharmacokinetic information, including the hyperechoic drug dispersion signal on EBUS and modelled intratumoral pressure gradients, could then be used to adapt the injection plan during the procedure. In this framework, AI would function as a closed-loop system: preprocedural imaging defines the initial plan, intraprocedural imaging updates spatial coverage, and post-procedural imaging or biomarker dynamics refine future delivery strategies [22,23].

AI may also contribute to platform development by comparing alternative delivery devices, predicting which catheter or needle configuration is most likely to achieve homogeneous tumour coverage, and identifying anatomical situations in which multi-site injection, microinjector-based circumferential delivery, or robotic-assisted access would be preferable. Such models could also incorporate safety constraints, including avoidance of highly vascular tumour regions, necrotic cavities, bronchial wall fragility, and regions where pressure injection may increase the risk of leakage into vascular or lymphatic channels. The clinical validation of AI-guided intratumoral navigation will therefore require more than demonstration of technical reach. Future studies should prospectively test whether AI-supported planning improves spatial drug coverage, reduces incomplete or excessive injection, standardises operator-dependent variability, and improves biologically meaningful endpoints such as immune-cell infiltration, circulating tumour DNA kinetics, and concordant response of injected and non-injected lesions. Validation should be performed in independent cohorts rather than in the training datasets used to develop the algorithms.

The single-session bronchoscopic paradigm—in which diagnosis, molecular staging, and intratumoral therapeutic delivery are performed in a single anaesthetic event—eliminates the 2–6 week interval between diagnostic bronchoscopy and treatment initiation that currently characterises standard oncological pathways. Population-based data demonstrate that each additional week of diagnostic delay is associated with a statistically significant reduction in overall survival in lung cancer; eliminating this interval through endobronchial single-session workflows would carry both clinical and patient quality-of-life benefits [10,15]. A pragmatic randomised trial comparing single-session versus two-stage bronchoscopic pathways—with time-to-treatment initiation as the primary endpoint and patient-reported outcomes as key secondary endpoints—would provide the evidence base for this operational innovation.

### 11.2. Rational Combinations, Next-Generation Platforms, and the Path to Translation

The mechanistic complexity of the tumour immune microenvironment—with its multiple, redundant immunosuppressive mechanisms—argues strongly against single-agent intratumoral approaches as the ultimate therapeutic strategy. Rational combination design—in which each component addresses a distinct, non-overlapping immunosuppressive mechanism—is a scientific priority. A factorial trial design permits estimation of the main effect of each agent and two-way interactions within a single trial; for a four-agent factorial design with two doses per agent, the minimum sample size for 80% power to detect main effects and interactions at α = 0.05 exceeds 400 patients. A platform trial design—a master protocol with a shared control arm and multiple experimental arms governed by adaptive randomisation based on interim biomarker and efficacy data—represents the most statistically efficient path to rational combination evaluation in this setting [11,21].

Three delivery platform innovations at the preclinical or early translational stage may reshape the technical landscape over the next decade. Aerosolised or inhalable delivery of therapeutic agents—demonstrated by Liu et al. [34] for inhalable EV-encapsulated IL-12 mRNA in orthotopic murine NSCLC—could extend therapeutic reach to peripheral lung nodules throughout the entire parenchyma simultaneously without bronchoscopic access. Closed-loop pharmacokinetic feedback, in which the computational drug distribution model [22] is updated during injection using real-time EBUS acoustic data, would transform intratumoral delivery from an open-loop into a precision-guided, closed-loop intervention. Living medicines—engineered T cells, NK cells, or stem cells producing therapeutic payloads continuously within the TME [60,61]—represent the convergence of cell therapy and intratumoral delivery that the next generation of trials is scientifically ready to explore.

Five specific commitments will determine whether endobronchial intratumoral therapy advances from biological optimism to clinical standard over the next decade: the randomised Phase II trial of IT therapy versus standard of care, adequately powered with embedded biomarker substudies; prospective external validation of AI-guided bronchoscopic navigation with pre-specified performance thresholds; standardisation of SBRT dosimetry and sequencing in combination with intratumoral delivery; expansion of clinical trial enrolment to LMIC populations through the tiered infrastructure model; and development of harmonised feasibility metrics enabling longitudinal tracking of equity progress [21,99]. These are not small commitments—they require funding, coordination, and the institutional willingness to design trials whose results may be negative. The science reviewed in this article provides a compelling foundation; what the next decade builds on that foundation will determine the clinical legacy of this field.

## 12. Conclusions

Endobronchial intratumoral delivery of immuno- and gene-based therapies represents a promising but still investigational strategy at the interface between interventional pulmonology, cancer immunology, and molecular medicine. The current evidence supports cautious translational inferences rather than definitive clinical conclusions: bronchoscopic platforms can provide precise access to central, hilar, mediastinal, and selected peripheral lung cancer lesions; local delivery can remodel the tumour immune microenvironment through mechanisms such as immunogenic cell death, cGAS–STING activation, CD8+ T-cell recruitment, and potential systemic immune priming; and these approaches are most likely to be clinically relevant when integrated with systemic immunotherapy, radiotherapy, or molecularly guided treatment rather than used as stand-alone interventions.

In the current NSCLC treatment landscape, the most plausible candidates are patients with advanced or recurrent disease, bronchoscopically accessible lesions, inadequate response or acquired resistance to immune checkpoint inhibitors, and tumour features suggesting immune exclusion or local immunosuppression. In such patients, bronchoscopy could eventually serve a dual diagnostic and therapeutic role, enabling tissue acquisition for molecular and immune profiling while delivering a local agent intended to restore or amplify systemic treatment sensitivity. Additional potential indications include oligoprogressive disease during otherwise effective systemic therapy and selected combinations with radiotherapy, particularly where local immune activation may enhance antigen release and systemic immune priming.

However, routine clinical adoption is not yet justified. Existing data remain dominated by Phase I trials, small prospective cohorts, case reports, and preclinical studies, and no completed randomised trial has demonstrated superiority over standard-of-care systemic therapy in NSCLC. Future development should therefore prioritise adequately powered prospective trials, standardised delivery protocols, validated biomarkers, longitudinal safety monitoring, and response criteria capable of accounting for immune-related pseudoprogression. Equitable implementation must also be considered, because these strategies require specialised bronchoscopy infrastructure, trained operators, imaging support, and multidisciplinary oncology pathways. If these conditions are met, endobronchial intratumoral therapy may become a precision locoregional adjunct capable of converting selected resistant lesions into immunologically actionable targets within multimodal NSCLC care.

## Figures and Tables

**Table 1:** Comparative procedural and translational characteristics of bronchoscopic platforms for intratumoral delivery in lung cancer.

Platform	Principal Lesion Target	Guidance Principle	Reported Diagnostic Performance	Therapeutic Reach	Key Translational Advantage	Main Limitations	Supporting References
Endobronchial ultrasound (EBUS)	Central, hilar, mediastinal, and peribronchial lesions or lymph nodes	Real-time curvilinear ultrasound-guided transbronchial needle placement	Mediastinal staging sensitivity approximately 88–93%	Central and peribronchial lesions; mediastinal or hilar nodal disease	Combines staging, tissue acquisition, molecular profiling, and local drug delivery in a single bronchoscopic procedure	Limited access to small peripheral lesions; depot injection may produce heterogeneous intratumoral distribution	[10,11,13,14]
Electromagnetic navigation bronchoscopy (ENB)	Peripheral pulmonary nodules with bronchoscopic pathway	CT-derived virtual airway roadmap with electromagnetic tracking	Diagnostic yield approximately 59–80%, depending on lesion size, CT bronchus sign, and registration accuracy	Peripheral lesions reachable through navigable airways	Extends bronchoscopic access beyond central airways and avoids direct transthoracic pleural traversal	Susceptible to CT-to-body divergence, respiratory motion, registration error, and reduced yield in small or bronchus-sign-negative lesions	[11]
Robotic-assisted bronchoscopy (RAB)	Peripheral and distal subsegmental lesions, including multifocal disease	Shape-sensing or robotic catheter navigation, frequently combined with cone-beam CT or fluoroscopic tool-in-lesion confirmation	Multisite real-world diagnostic sensitivity approximately 85% for peripheral lesions	Peripheral, distal, and multifocal pulmonary lesions within a single anaesthetic session	Improved catheter stability, access to distal lesions, and potential for multi-site therapeutic delivery	High platform cost, learning-curve effects, need for advanced imaging support, and limited accessibility outside specialised centres	[15,18]

Abbreviations used in this table are defined in the Abbreviations section.

**Table 2:** Viral and non-viral intratumoral delivery systems: comparative properties.

Delivery System	Cargo (kb)	Immunogenicity	Repeat Dosing	Clinical Stage	Key Advantage	Main Limitation
Viral vectors						
Adenovirus (Ad5)	~36 kb	High (capsid proteins)	Limited (NAb)	Phase I-III (NSCLC: GMCI, MEM-288)	High transduction efficiency	Pre-existing NAb in ~50% adults
AAV (serotype 5/6)	~4.7 kb	Low-moderate	Better tolerated	Phase I-II (lung gene therapy)	Long-term expression (months)	Small cargo; pre-existing immunity
Oncolytic virus (OV)	Variable	Moderate (ICD-mediated)	Possible with modified capsid	Phase I (MEM-288 NCT05076760; RT-01 NCT05205421)	Dual: cytolysis + immunostimulation	Immune neutralisation over time
Non-viral systems						
LNP-mRNA	Unlimited	Low-moderate (innate, dose-dep.)	Yes (transient expression)	Phase I (mRNA-2752 NCT03739931)	Programmable; fast manufacturing	Transient expression (24–72 h)
PLGA nanoparticles	Unlimited	Very low	Yes	Phase II (NanoPac NCT04314895)	Sustained release; biodegradable	Lower transfection efficiency
PEGylated peptide (siRNA)	Small (siRNA)	Very low	Yes	Preclinical (Man et al.)	Co-delivery EGFR + PD-L1 siRNA	In vivo stability; off-target
Extracellular vesicles (EV)	Small-moderate	Minimal	Yes	Preclinical (Liu et al.)	Inhalable; biocompatible	Manufacturing scalability
CRISPR-Cas9 (LNP)	~4–6 kb (RNP or mRNA)	Low-moderate	Challenging (immune to Cas9)	Preclinical (Lentsch et al.)	Permanent gene editing; precise	In vivo efficiency; Cas9 immunity

Abbreviations used in this table are defined in the Abbreviations section. Clinical stage data are based on the trials and studies referenced in the table and bibliography.

**Table 3:** Regulatory pathways for cell and gene therapy products by jurisdiction.

Jurisdiction	Regulatory Body	Key Pathway/Designation	Main Requirement	Lag vs. FDA	2024 Example
United States	FDA	RMAT (Regenerative Medicine Advanced Therapy); BLA/NDA	Clinical POC; rolling review; adaptive trial design	Reference (0)	Lovo-cel (sickle cell)
European Union	EMA	ATMP classification; CAT scientific review; centralised procedure	GMP manufacturing; long-term follow-up ≥15 yr	12–24 months	Aucatzyl (ALL)
China	NMPA	Conditional approval in oncology; priority review	Domestic clinical data increasingly required	18–36 months	CT053 (myeloma)
Japan	PMDA	Sakigake designation (breakthrough); early approval	Confirmatory data post-approval permitted	12–24 months	Kimriah (paediatric ALL)
UK (post-Brexit)	MHRA	Innovative Licensing and Access Pathway (ILAP)	Rolling review; NHS England parallel engagement	6–18 months	Casgevy (sickle cell/beta-thal)
LMIC (no authority)	Reference to HIC SRA	WHO prequalification (emerging); reference to FDA/EMA	Waiver or reliance mechanism needed	+2–5 yr (if any)	Access rarely achieved

Abbreviations used in this table are defined in the Abbreviations section. Approval lag refers to the typical delay from FDA approval to local approval. The 2024 examples are based on Jian et al. [62].

**Table 4:** Comparative performance characteristics of predictive and prognostic biomarkers for systemic response to immunotherapy in advanced NSCLC.

Biomarker	Clinical Threshold	AUC for ORR (Approx.)	HR for OS (95% CI)	Sensitivity at Threshold	Specificity at Threshold	Threshold Derivation
TMB	≥10 mut/Mb (FDA)	0.64–0.68	0.58 (0.43–0.78)	57–65%	63–70%	Youden Index on ROC (KEYNOTE-158, post hoc)
PD-L1 TPS ≥ 50%	≥50% (pembrolizumab 1L NSCLC)	0.67–0.72	0.50 (0.37–0.68) †	64–68%	70–74%	Pre-specified (KEYNOTE-024)
PD-L1 TPS ≥ 1%	≥1% (broad ICI eligibility)	0.58–0.63	0.69 (0.56–0.85)	80–85%	30–40%	Pre-specified (multiple trials)
CD8+ TILs	High vs. Low (median/tertile)	Not established	0.61 (0.48–0.77) ‡	—	—	Exploratory; varies by study
Tertiary lymphoid structures (TLS)	Present vs. Absent (H&E)	Not established	0.59 (0.48–0.72) §	—	—	Binary classification
ctDNA (dynamic Δ)	≥50% decrease at 6 weeks	0.74–0.80	0.44 (0.29–0.67) ‖	72–78%	78–83%	Exploratory optimal cutpoint
Platelet PD-L1	Elevated vs. baseline (continuous)	0.72 (0.63–0.81)	0.51 (0.38–0.68)	69%	74%	Continuous (AUC-derived)

Abbreviations used in this table are defined in the Abbreviations section. Notes: † KEYNOTE-024 OS data, pembrolizumab versus chemotherapy in PD-L1 ≥50% NSCLC [78]. ‡ Pooled estimate from meta-analysis of CD8+ TIL density and OS in NSCLC [79]. § TLS meta-analysis, 17 studies, 4291 patients [74]. ‖ Estimate from methylation-based ctDNA and longitudinal tracking cohorts [80,81]. All AUC values are approximate and derived from single-arm or observational cohorts; they are therefore subject to optimistic bias in the absence of independent external validation.

**Table 5:** Summary of clinical trials and prospective cohort studies of bronchoscopic intratumoral delivery in lung cancer, organised by therapeutic category.

Trial/NCT	Phase	Agent/Platform	N	Primary Endpoint	Key Efficacy/Biological Signal	Refs
EBUS-Guided Intratumoral Chemotherapy						
EBUS-TBNI cisplatin (Mehta and Jantz)	I/feasibility	Cisplatin IT via 22G EBUS needle	Series	Safety; tumour devitalisation	Tumour devitalisation in mediastinal/hilar recurrences post-RT; proof-of-concept for EBUS-guided IT drug delivery	[86]
EBUS-TBNI cisplatin TME study (DuComb et al.)	Prospective cohort	Cisplatin IT via EBUS-TBNI; repeat sessions	Prospective	Immune microenvironment changes (CD8+ TIL density)	Median 3.2-fold ↑ CD8+ TIL density post-treatment (*p* = 0.012); PD-L1 upregulation; myeloid modulation; no systemic DLTs	[87]
Endobronchial IT Chemotherapy Multicenter Pilot (Yarmus et al.)	I multicentre	Nanoparticulate paclitaxel via Blowfish catheter	19 treated	Safety/feasibility	0/19 device-related SAEs; 0 ADEs; mean 3.4 injections/session; upper 95% CI AE rate (Clopper-Pearson) = 17.6%	[19]
Gene Therapy—Intratumoral Viral Vectors						
Ad-p53 IT + cisplatin (Nemunaitis et al.)	I	Adenoviral wild-type p53 IT; cisplatin IV (sequential)	25	Safety; MTD of Ad-p53	ORR 8% (95% CI 1.0–26.0%); DCR 40% (95% CI 21.1–61.3%); transgene expression dose-dependent; no p53-related systemic toxicity	[47]
GMCI Phase I neoadjuvant (Predina et al.)	I neoadjuvant	Ad-HSV-TK IT + ganciclovir IV; surgical resection Day 28	Resectable NSCLC	Safety; immune activation (CD8+ TIL density)	CD8+ TIL fold-change 3.2 (*p* = 0.012, Wilcoxon); granzyme B ↑; PD-L1 ↑; rationale for IT + ICI combination established	[54]
GMCI 5-year outcomes (Aicher et al.)	I/II long-term	Ad-HSV-TK IT + ganciclovir (neoadjuvant); resection	Resectable NSCLC	Long-term OS; late adverse events	Favourable 5-yr OS vs. historical controls; 0 late vector-related AEs; 0 insertional mutagenesis; 0 secondary malignancies at 60 months	[55]
Dendritic Cell Vaccines—Intratumoral						
IT CCL21-gene-modified DC (Lee et al.)	I	Ad-CCL21-transduced autologous DC; IT injection	NSCLC	Safety; immune biomarker changes	CD8+ TIL ↑ (dose-dependent); IFN-γ ↑ in TME; PD-L1 ↑ post-treatment; no systemic CRS	[44]
Ad-CCL21-DC + pembrolizumab (NCT03546361)	I/II	Ad-CCL21-DC IT + pembrolizumab IV	Stage IV NSCLC	Safety; ORR	Ongoing; rationale: CCL21-DC converts PD-L1-negative TME to PD-L1-positive, sensitising tumour to ICI	[45]
Neoantigen-targeted DC vaccination (Ingels et al.)	I/II	Neoantigen-loaded DC; IT/SC injection	Advanced NSCLC	Safety; T-cell response; PFS	TCR clonotype expansion at 12 months; TIL infiltration in post-treatment biopsies; median PFS 8.6 months (single-arm, no comparator)	[43]
mRNA-LNP—Intratumoral Immunostimulation						
mRNA-2752 IT ± durvalumab (NCT03739931)	I dose-escalation	mRNA-2752 LNP (OX40L/IL-23/IL-36γ) IT; ±durvalumab IV	Advanced solid tumours incl. NSCLC	Safety; DLT; MTD; pharmacodynamics	ISG upregulation confirmed in paired biopsies; immunostimulatory TME gene signature induced; no DLTs at first two dose levels reported	[32,33]
Oncolytic Virotherapy—Intratumoral						
MEM-288 IT ± SOC (NCT05076760)	I	MEM-288 adenoviral (IFNβ + CD40L) IT; ±SOC	Advanced solid tumours + NSCLC expansion	Safety; DLT; MTD; anti-tumour activity	Ongoing; NSCLC expansion cohort enrolling; no DLTs at Dose Levels 1–2; dual innate + adaptive activation mechanism	[39]
RT-01 oncolytic virus (NCT05205421)	I	RT-01 oncolytic virus IT	Extensive-stage SCLC	Safety; preliminary efficacy in SCLC	Ongoing; target population chosen for high TMB and initial ICI responsiveness	[40]
STING Agonists						
E7766 IT (INSTAL-101) (Luke et al.)	I/Ib	E7766 STING agonist IT; dose escalation	Advanced solid tumours	Safety; DLT; pharmacodynamics	STING pathway target engagement confirmed; IFN-stimulated gene signature ↑; objective responses rare	[67]
Dazostinag + pembrolizumab + RT (Cooper et al.)	Ib	Dazostinag (TAK-676) IV + pembrolizumab IV + hypofractionated RT	Advanced NSCLC, TNBC, SCCHN	Safety; pharmacodynamics; preliminary ORR	Systemic STING activation with abscopal immune signals in ICI-refractory NSCLC	[66]
Non-Viral Nanoparticle—Intratumoral						
NanoPac IT injection (NCT04314895)	II	Nanoparticulate paclitaxel IT; repeat 28-day cycles	Advanced NSCLC (inoperable)	Tolerability (≥2 cycles); secondary: ORR, local control	Completed 2023; tolerability profile consistent with IV paclitaxel; no procedure-related SAEs at interim; full efficacy data pending	[88]

Abbreviations used in this table are defined in the Abbreviations section. ↑ indicates an increase relative to baseline (pre-treatment).

## Data Availability

No new data were created or analyzed in this study. Data sharing is not applicable to this article.

## Abbreviations

The following abbreviations are used in this manuscript:

AAV	Adeno-associated virus
ADE	Adverse device effect
AE	Adverse event
AI	Artificial intelligence
ALL	Acute lymphoblastic leukaemia
ATMP	Advanced therapy medicinal product
ATP	Adenosine triphosphate
AUC	Area under the receiver operating characteristic curve
BED	Biological effective dose
BLA	Biologics licence application
CAT	Committee for Advanced Therapies
CBCT	Cone-beam computed tomography
CD8+	CD8-positive cytotoxic T lymphocyte
CD73	5′-ectonucleotidase
cGAS	Cyclic GMP-AMP synthase
CI	Confidence interval
CNN	Convolutional neural network
CRISPR	Clustered regularly interspaced short palindromic repeats
CT	Computed tomography
ctDNA	Circulating tumour DNA
DAMPs	Damage-associated molecular patterns
DC	Dendritic cell
DCR	Disease control rate
DLT	Dose-limiting toxicity
EBUS	Endobronchial ultrasound
EBUS-TBNA	Endobronchial ultrasound-guided transbronchial needle aspiration
EBUS-TBNI	Endobronchial ultrasound-guided transbronchial needle injection
EGFR	Epidermal growth factor receptor
EMA	European Medicines Agency
ENB	Electromagnetic navigation bronchoscopy
EV	Extracellular vesicle
FDA	US Food and Drug Administration
FDR	False discovery rate
GGTI	Global Gene Therapy Initiative
GMCI	Gene-mediated cytotoxic immunotherapy
GMP	Good manufacturing practice
GOx	Glucose oxidase
H&E	Haematoxylin and eosin
HIC	High-income country
HMGB1	High-mobility group box 1 protein
HR	Hazard ratio
HSV-TK	Herpes simplex virus thymidine kinase
ICD	Immunogenic cell death
ICH	International Council for Harmonisation
ICI	Immune checkpoint inhibitor
ICMRA	International Coalition of Medicines Regulatory Authorities
IFN	Interferon
IFN-γ	Interferon gamma
IL	Interleukin
ILAP	Innovative Licensing and Access Pathway
irAE	Immune-related adverse event
iRECIST	Immune-modified Response Evaluation Criteria in Solid Tumours
ISG	Interferon-stimulated gene
IT	Intratumoral
KRAS	Kirsten rat sarcoma viral proto-oncogene
LIC	Low-income country
LMIC	Low- and middle-income country
LNP	Lipid nanoparticle
MDSC	Myeloid-derived suppressor cell
MHC	Major histocompatibility complex
MHRA	Medicines and Healthcare products Regulatory Agency
mRNA	Messenger ribonucleic acid
MTD	Maximum tolerated dose
NAb	Neutralising antibody
NCT	National Clinical Trial identifier
NDA	New drug application
NMPA	National Medical Products Administration
NSCLC	Non-small-cell lung cancer
OR	Odds ratio
ORR	Objective response rate
OS	Overall survival
OV	Oncolytic virus
PD-1	Programmed death-1
PD-L1	Programmed death-ligand 1
PEG	Polyethylene glycol
PET/CT	Positron emission tomography/computed tomography
PFS	Progression-free survival
PLGA	Poly(lactic-co-glycolic acid)
PMDA	Pharmaceuticals and Medical Devices Agency
POC	Proof of concept
qPCR	Quantitative polymerase chain reaction
QALY	Quality-adjusted life-year
RAB	Robotic-assisted bronchoscopy
RECIST	Response Evaluation Criteria in Solid Tumours
RMAT	Regenerative Medicine Advanced Therapy designation
RMST	Restricted mean survival time
RNP	Ribonucleoprotein
ROC	Receiver operating characteristic
RT	Radiotherapy
SAE	Serious adverse event
SBRT	Stereotactic body radiotherapy
SCLC	Small-cell lung cancer
siRNA	Small interfering RNA
SOC	Standard of care
SRA	Stringent regulatory authority
STING	Stimulator of interferon genes
TCR	T-cell receptor
TGF-β	Transforming growth factor beta
TIL	Tumour-infiltrating lymphocyte
TLS	Tertiary lymphoid structure
TMB	Tumour mutational burden
TME	Tumour microenvironment
TPS	Tumour proportion score
TREX1	Three-prime repair exonuclease 1
UMIC	Upper-middle-income country
WHO	World Health Organization
